# Survey of Midbrain, Diencephalon, and Hypothalamus Neuroanatomic Terms Whose Prosomeric Definition Conflicts With Columnar Tradition

**DOI:** 10.3389/fnana.2019.00020

**Published:** 2019-02-27

**Authors:** Luis Puelles

**Affiliations:** Departamento de Anatomía Humana y Psicobiología, IMIB-Arrixaca Biomedical Institute, University of Murcia, Murcia, Spain

**Keywords:** columnar model, prosomeric model, neuroanatomical advances, novel anatomic terms, forebrainterminology, forebrain axis, lamina affixa, thalamo-striatal sulcus

## Abstract

Recent neuroanatomic concepts and terms referring to the non-telencephalic forebrain are presented and discussed, in context with the present scenario in which the old columnar paradigm is being substituted by the prosomeric model, largely on the basis of novel molecular and experimental evidence.

“Since some variety, including that of terminology and spelling, may be regarded as the ‘spice of life,' I nevertheless prefer to write ‘piriform' [instead of ‘pyriform] without prejudice to the preference of others”

Kuhlenbeck (1973).

(The Central Nervous System of Vertebrates, Vo l3., Part II., footnote 289, p.668).

## Introduction

**Figure 1:**
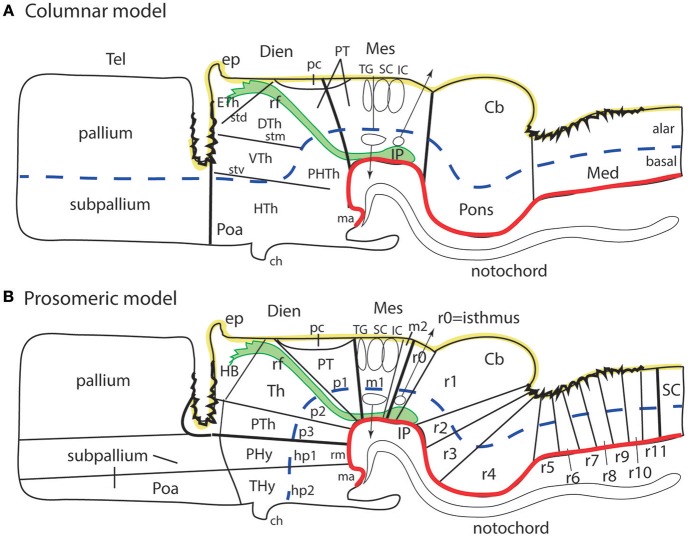
Schematic comparison of the columnar and prosomeric models (original drawing). The same basic drawing shows in both cases telencephalon (Tel), diencephalon (Dien), mesencephalon (Mes), cerebellum (Cb), pons, and medulla (Med), as well as the notochord, the floor plate (as defined molecularly and by a glial palisade; in red), the roof plate inclusive of chorioidal plexi yellow background with black irregular domains, and the alar-basal boundary (thick blue dash line), which divides the alar and basal longitudinal zones. The retroflex tract descending from the habenula to the interpeduncular nucleus (rf, IP; green) is also depicted. Differences between the two schemata refer to the boundaries limiting large regions one from another (thick black lines) and boundaries separating subdivisions (columns or neuromeres), representing either ventricular sulci or ridges (thin black lines). **(A)** Columnar model: Here I used the recent version of the columnar model used by Swanson (2012), because he reasonably accepts a bending of the alar-basal boundary around the cephalic flexure (see blue dash line and its curve parallel to the floor in red); this axis landmark later ascends arbitrarily in front of the ventral thalamus (VTh) into the telencephalon, separating there pallium from subpallium. Note this model includes a tegmental posterior hypothalamus that reaches the midbrain next to the retroflex tract (PHTh), and expands rostrally into the standard hypothalamus (HTh). This model divides the hindbrain merely into medulla and cerebello-pontine complex. The midbrain is larger in this model, because it encompasses isthmic and prepontine formations caudally (including the trochlear nucleus/nerve and the interpeduncular complex; IP), and pretectal formations rostrally (caudal half of pretectum rostral to the tectal gray (TG), and the parvocellular red nucleus -not shown). Note the rostral midbrain limit passes through the middle of the posterior commissure (pc) and is not strictly transversal (= outdated His (1893) limit; compare Figure 2A). This model does not postulate a specific limit between the pretectum and the dorsal thalamus and epithalamus, but other columnar sources accept it passes behind the retroflex tract (rf). The columnar diencephalic subdivisions show parallel sulci thalami medius and ventralis (stm, stv) which delimit HTh, VTh, and DTh. The std (sulcus thalami dorsalis) separates DTh from epithalamus (ETh), but it does not course parallel to the others. The topological relationship of these sulci relative to the axial landmark (blue dash line) is variable: the std is parallel to it, thus being the only truly longitudinal diencephalic sulcus in this model; the stm is orthogonal to the axis, while the stv can be seen as parallel to the axial reference ascending into the telencephalon, or as orthogonal to the sulcus limitans. Inconsistently with the supposed longitudinal nature of the DTh and VTh columns, the schema shows that they reach the roof plate at one end and point into the floor plate at the other end. This is why authors such as Kappers (1947) interpreted these “columns” as transversal domains (see Figure 6A). The hypothalamus extends beyond the rostral end of the epichordal floor plate at the mamillary body, so that it needs *ad hoc* causal underpinnings for justifying the implied more rostral extent of dorsoventral patterning. **(B)** Prosomeric model: All the neuromeric units are included, highlighting their regular topology with regard to the floor plate (red), the alar-basal boundary (blue), and the roof plate (yellow; note the roof plate extends farther in the telencephalon, along the commissural septum, finally building a roof for the preoptic area (Poa) at the anterior commissure level; this telencephalic roof relationship is also incongruent with the columnar axial concept). The prosomeric model recognizes many more subdivisions in the brainstem, and notably ascribes the pons (r2-r4) to different rhombomeres than the cerebellum (r0, r1), as indicated by fate mapping. The prepontine hindbrain (r0, r1) is thus distinguished from the midbrain, which consequently results reduced in size and contents. The rostral midbrain limit passes behind the posterior commissure. Note the interpeduncular complex now lies in the prepontine hindbrain (IP). The m2 mesomere represents the novel preisthmus concept. As regards the diencephalon, it can be easily seen that basically the same regions are interpreted in a different and more solid topologic framework supported by gene expression patterns. There appears a diencephalic tegmental (basal) region, which contains part of the mesodiencephalic substantia nigra and ventral tegmental area. The hypothalamic floor is restricted to retromamillary and mamillary subdomains (rm, ma). The entire forebrain complex, from secondary prosencephalon to caudal midbrain, is divided into alar and basal territories.

**Figure 2:**
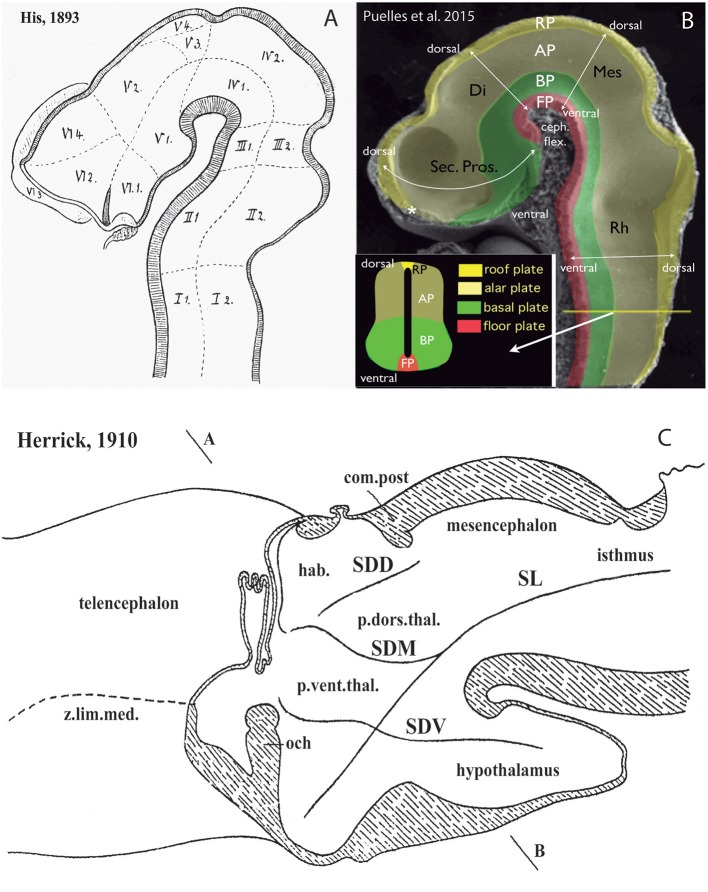
Longitudinal vs. transversal neural tube directions in the conceptions of His (1893) **(A)**; Martínez et al. (2012) and Puelles et al. (2015) **(B)**; and Herrick (1910) **(C)** (no copyright permission required). **(A)** The pioneering view of His (1893) illustrates his original notion of axial longitudinal zones (floor, basal, alar, and roof plates) found throughout the brain; the common alar-basal boundary coincides with his *sulcus limitans*, which represents heterochronic differential neurogenesis prevalent at the basal plate (the sulcus is formed in early human embryos due to intraventricular bulging of the neurogenetically precocious basal plate, i.e., the set of domains I-VI numbered 1; the remaining domains represent the alar plate). A marked cephalic flexure is represented, and the axial landmark zones (floor, basal, alar, and roof plates) all bend around it, indicating a *bent* brain length axis. Theoretically transversal limits between the domains I–VI are also marked. Note the definition of an isthmic segment at the rostral end of the hindbrain (identified as III1 + III2). The midbrain (IV1 + IV2) appears delimited from the diencephalon *sensu stricto* (V1–V4) by a tentative oblique plane (later non-corroborated) that jumps from the middle of the posterior commissure to the mamillary body neighborhood. The hypothalamus was first defined by His in this schema as the sum of the V1 and VI1 domains, both entirely within the basal plate. The boundary separating V from VI has later been validated for the alar domains, but not for the basal ones. V1 underlies the alar “thalamus” and “epithalamus” (thalamic hypothalamus), while VI1 underlies the preoptic recess (optic hypothalamus) as well as the striate and parolfactory bodies (subpallium; VI2, VI3); the subpallium was accordingly held to reach the rostral part of the alar-basal boundary, at the preoptic recess (VI4 represents the telencephalic pallium). **(B)** This image, extracted from book chapters published in 2012 and 2015, shows the prosomeric assumptions about the longitudinal organization of the neural tube in a mouse embryo, which follow closely the model of His. The only difference is that the longitudinal zones are defined by primary early gene markers (rather than secondary differentiation patterns) and the rostral end of the alar-basal limit ends under the prospective optic chiasma, rather than at the preoptic recess (the sulcus limitans only approximates the primary (molecular) alar-basal boundary, due to its tertiary growth-related nature). In this image the floor (FP) is red, the basal plate (BP) green, the alar plate (AP) light yellow, and the roof plate (RP) strong yellow (see also the explanatory inset, a cut at spinal cord level); an asterisk marks the roof's rostral end at the prospective anterior commissure. White arrows indicate the changing dorsoventral dimension due to the cephalic flexure of the brain axis. The floor ends rostrally at the mamillary pouch (correlative with an initial chordal induction and the early position of the notochordal tip). The rostral neural line extending dorsoventrally from the asterisk (roof) to the mamillary body (floor) represents the novel prosomeric notion of “acroterminal area.” **(C)** Modified drawing showing the alternative columnar model of Herrick (1910), as defined in an adult urodele. This initial study of Herrick still admitted the sulcus limitans of His (SL, compare with **A,B**), but no longer depicted it as closely following in curvature the cephalic flexure; it was implied not to represent an axial landmark, and was wholly disregarded in subsequent work. The axial landmark role was assigned to the sulcus diencephali medius (SDM) and sulcus diencephali ventralis (SDV) (otherwise also known as “thalamic” sulci), arbitrarily held to separate “longitudinal columns' identified as hypothalamus, pars ventralis thalami (p.vent.thal.) and pars dorsalis thalami (p.dors.thal.). An additional sulcus nearly orthogonal to the SDM, which separates the habenular region or epithalamus (hab.) from the p.dors.thal, was taken as sulcus diencephali dorsalis (SDD). Note the SDD finishes roughly under the posterior commissure (com.post.), thus implying that the pretectum (not identified) was half epithalamic and half dorsal thalamic. Remarkably, both SDM and SDV are clearly orthogonally disposed relative to the sulcus limitans, as well as to the forebrain roof and floor plates (check also **A,B**), and they are not continuous either with the midbrain or with the telencephalon. Their topology with regard to the cephalic flexure is vaguely represented.

**Figure 3:**
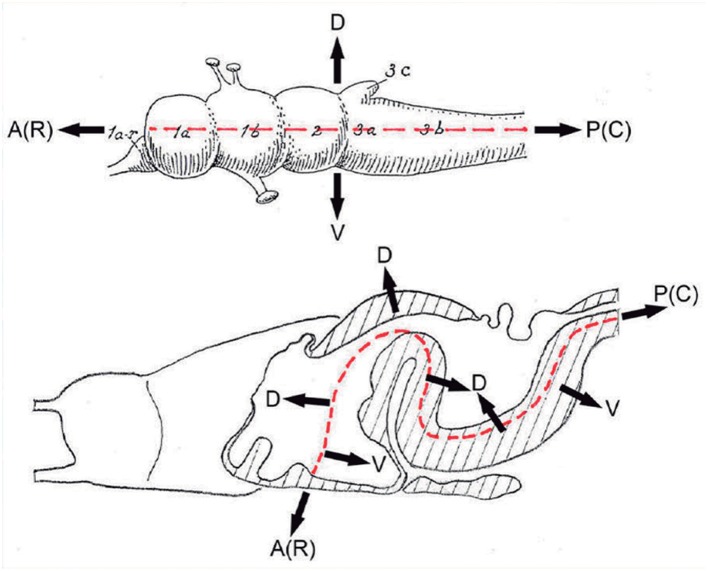
These two schemata are copied from Nieuwenhuys and Puelles (2016) (plate 50) (no copyright permission required). They highlight the crucial difference in the axial morphological reference for the brain used by columnar authors as compared to neuromeric authors following His (1893). The upper *columnar* schema was drawn by Ranson (1928), and it pretended to illustrate how the length axis of a primitive brain (red dashes) passes straightly through the telencephalon (1a), the diencephalon (1b), the midbrain (2), and the hindbrain (3a,3b; 3c is the cerebellum). The tags A(R) and P(C) refer to anterior (rostral) and posterior (caudal), respectively. D and V mark the orthogonal dorsoventral dimension. Remarkably, the cephalic flexure is not represented, though it appears in all vertebrates (an instance of psychological negation). The lower schema represents a gymnophionan (amphibian) brain whose cephalic flexure is extremely marked. The length axis (red dashes) is marked according to *prosomeric* tenets following the observable curvature and ending behind the optic chiasma (the telencephalon is understood as a dorsal outgrowth of the hypothalamic alar plate). As in the upper schema, the AP course of the axis decides what is dorsal (D) or ventral (V).

**Figure 4:**
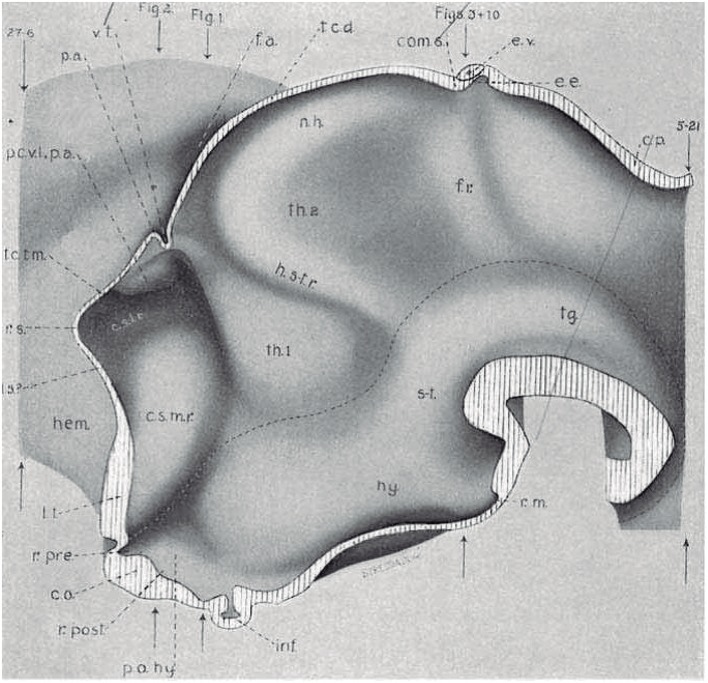
Copy of a reconstruction of the ventricular surface of a 19 mm human embryo published by Bailey (1916) (no copyright permission required). The axial reference is the sulcus limitans of His, an untagged dash line bending around the cephalic flexure and ending rostrally at the preoptic recess (r.pre). Modern versions of this limit, informed by neuronal differentiation markers and genoarchitectonic markers, instead make it end rostrally at the postchiasmatic recess (r.post), thus allowing the eye vesicles and the optic chiasma (c.o.) to represent alar structural elements. Kuhlenbeck proposed another variant with a rostral end of sulcus limitans at the mamillary recess (r.m.; reviewed in Kuhlenbeck, 1973); this option would ascribe the tuberal hypothalamus to the alar plate, but runs counter to early tuberal expression of the *Shh* gene. The standard basal plate zone delimited by the sulcus limitans (check Figures 2A,**B**) carries successively the tags for tegmentum (tg; at pretectal and midbrain levels), subthalamus (s-t; at retromamillary level) and hypothalamus (hy; at tuberal level). Bailey (1916) departs here from the original (His, 1893) notion, which completely equates the old subthalamus of Forel (1877) with his hypothalamus (Figure 2A). The alar plate region shows two compartments identified as th.1 and th.2, referring to two thalamic regions; according to the columnar model of Herrick (1910), these would be identified respectively as ventral and dorsal thalamus, while in the columnar model they represent the prethalamus and thalamus, respectively. However, the two boundaries that limit the thalamus (dorsal thalamus) are not “longitudinal' sulci, but are identified by Bailey (1916) as *transversal ridges* that converge into the cephalic flexure. Caudally there is a ridge caused by the retroflex tract (f.r.), which we now know courses at the limit between the thalamic and pretectal diencephalic prosomeres (see Figures 1B, 5, 6); rostrally another transversal ridge (identified as the habenulo-subthalamic ridge, h.s-t.r.) extends from the roof into the sulcus limitans, roughly pointing to the basal area identified as “subthalamus.” This ridge corresponds to the zona limitans intrathalamica of Rendahl (1924) and Gilbert (1935). The prethalamus (ventral thalamus) area is limited rostrally by a shallow sulcus that might correspond to Herrick's sulcus diencephali ventralis. It does not extend beyond the sulcus limitans and corresponds to what more modern columnar authors have identified as sulcus hypothalamicus of Monro, the supposed continuation of a partially bent columnar axis into the telencephalon (Figure 1A). This so-to-speak “innocent” reconstruction done outside of any school shows that the same morphology has been interpreted as “longitudinal” or as “transversal” depending of the axis accepted by the authors.

**Figure 5:**
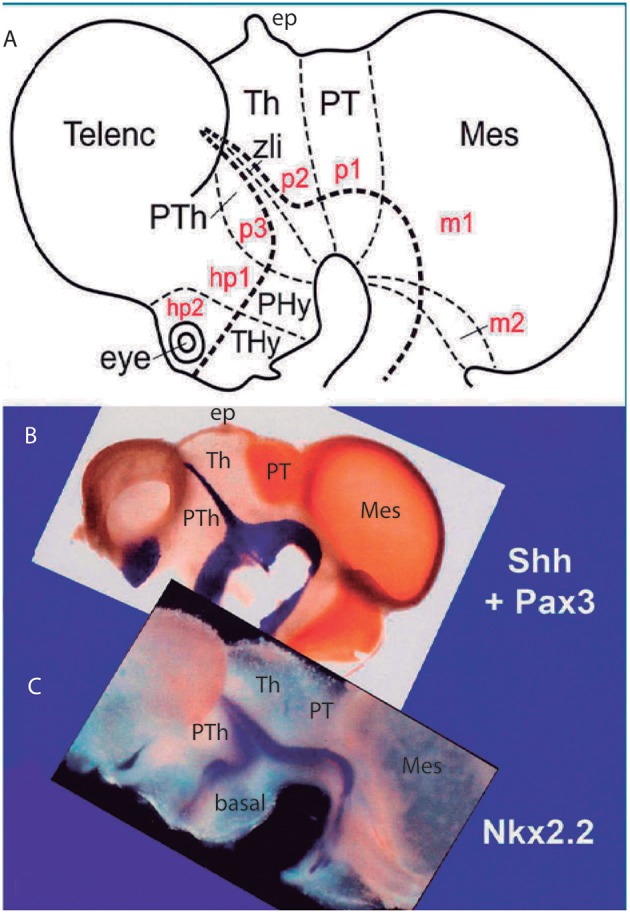
Original images from chick embryo brains illustrating molecular support for the longitudinal axial landmarks postulated in the prosomeric model (no copyright permission required). **(A)** Schematic view of the prosomeric forebrain, with the mesencephalic m1 and m2 mesomeres, the diencephalic pretectal, thalamic and prethalamic prosomeres p1–p3, and the hypothalamo-telencephalic prosomeres hp1 and hp2. The molecular alar-basal boundary curves around the cephalic flexure and associates to an orthogonal spike limiting the thalamus (Th; alar p2) from prethalamus (PTh, alar p3). This spike is known since Rendahl (1924) as the zona limitans intrathalamica (zli), which is understood nowadays as a *mid-thalamic secondary organizer* that releases diffusible SHH and WNT signals contributing to inner regionalization of Th and PTh, possibly also of pretectum (PT) (see Puelles and Martinez, 2013). The midbrain is organized instead by FGF8 signals spreading from the *isthmic organizer*, found just caudal to m2 (preisthmus) (review in Puelles, 2017). The *peduncular* and *terminal* segmental parts of the hypothalamus are also identified (PHy, THy); the corresponding hypothalamo-telencephalic prosomere hp1 extends into the evaginated telencephalic hemisphere, while hp2 ends in the subpallial preoptic area (unmarked). **(B)** Wholemount of a chick embryo double reacted for *Shh* mRNA *in situ* hybridization (blue signal) and immunoreaction against the protein transcription factor coded by *Pax3* (brown signal). The Shh signal clearly delineates the floor and basal longitudinal zones of the whole forebrain (secondarily there appears a downregulation of this signal at the tuberal hypothalamus). The zli (compare **A**) shows its *Shh*-positive core, which gives it its anteroposterior signaling capacity as a secondary organizer; the transverse zli spike connects ventrally with the similarly Shh-positive basal plate (different genomic enhancers are implied, so that the zli is not an extension of the basal plate); there is additional separated Shh expression at the preoptic area of the subpallium. On the other hand, *Pax3* signal is characteristic of a dorsal part of the pretectal alar plate and corresponding roof plate (there is also selective expression at the thalamic roof plate). This pattern gives partial molecular support to the interneuromeric thalamo-pretectal boundary, in lying just caudal to the retroflex tract (but leaves a ventral part of the pretectal alar plate negative). Moreover, extensive Pax3 signal appears likewise at the alar region of the prepontine hindbrain (behind the isthmic constriction; note the Shh signal marks here only the floor plate, and the hindbrain basal plate is unlabeled), and at the alar midbrain. Both the zli, separating p3 and p2, and the thalamo-pretectal border (p2/p1) are transversal limits that are distinctly orthogonal to the longitudinal basal plate and the underlying cephalic flexure. **(C)** Wholemount of a chick embryo reacted for Nkx2.2 *in situ* hybridization (blue signal). This gene marker is upregulated exclusively across the border of *Shh* expression by particularly high levels of diffusing SHH protein. We see accordingly signal as a band that follows the alar-basal border seen in **(B)**, and also climbs up and down the spike of the zli core domain expressing likewise *Shh*. Note this combined *Shh* and *Nkx2.2* expression pattern is continuous through the whole forebrain, from midbrain to hypothalamus, and does not enter into the telencephalon! Moreover, it cuts the hypothalamus into alar and basal moieties (leaving the optic stalk on the alar side), contrary to columnar assumptions. This pair of genes is expressed slightly differently in the hindbrain, namely across the floor-basal boundary, due to the local restriction of Shh to the floor plate. This patterning difference between forebrain and hindbrain corroborates the modern isthmic boundary of the midbrain, as well as the ascription of midbrain to the forebrain. Such patterns as shown here in **(B,C)**, with more gene markers added, is what is meant with the expression “primary molecular definition of a brain boundary”: a set of coherent gene or protein expression patterns that demonstrate collectively the reality and precise position of neuroepithelial boundaries before neurogenesis occurs, underpinning differently fated neural wall regions (as corroborated experimentally), and pointing to the implied causal mechanistic correlations. These limits precede neurons in the mantle, though they later overlap with their architectonic boundaries; they accordingly condition by their differential regulatory functions the distinct histogenetic secondary phenomena that occur subsequently at each side of these boundaries; these limits invariably finish as more or less visible adult brain boundaries, and rarely coincide with ventricular sulci (sometimes experimental methods are needed to visualize them at postnatal stages).

**Figure 6:**
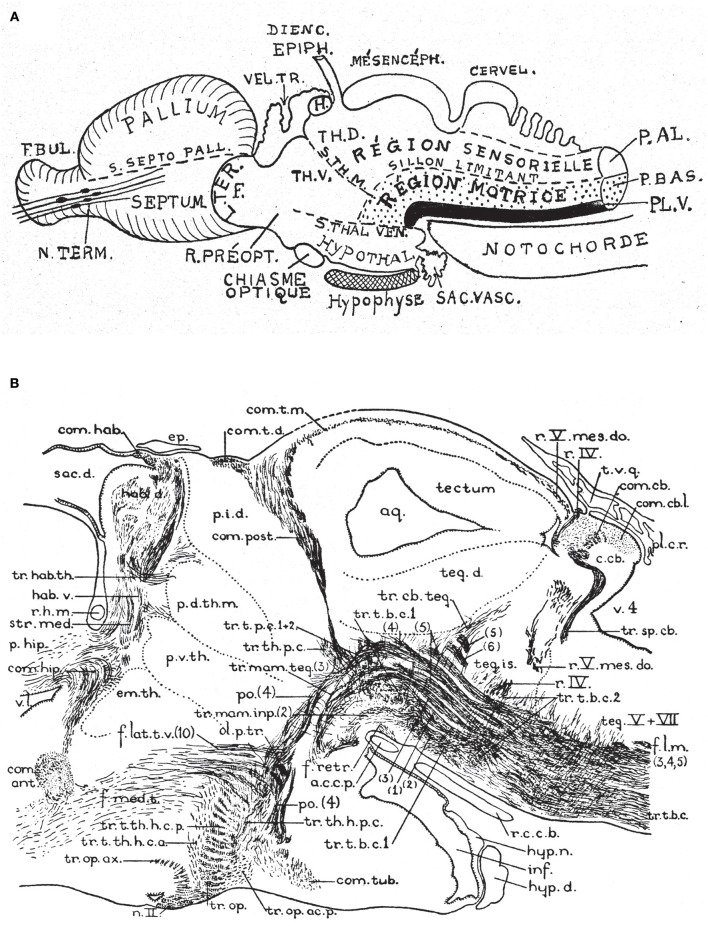
Two classic images showing evidence that supposed longitudinal diencephalic columns actually represent transversal forebrain domains (no copyright permission required). **(A)** Schematic drawing of a primitive anamniote brain by Kappers (1947) that includes on one hand the basic floor, basal, alar and roof longitudinal zones of His (1893) (region sensorielle = alar plate; region motrice = basal plate; sillon limitante = sulcus limitans of His; the floor plate is in dense black; note its rostral end is roughly correlated with the tip of the notochord). Herrick's (1910) thalamic sulci stm and stv that limit the DTh, VTh, and HTh columns are also represented. Kappers here clearly implies that he thinks that these sulci and “columns” are transversal, since he draws them orthogonal to the longitudinal zonation system. **(B)** This drawing is a very realistic rendering by Herrick (1936) of a fiber-stained sagittal section through the brain of *Amblystoma* (an urodele). Note a cephalic flexure is distinctly present, and longitudinal tracts coursing from the brainstem into the forebrain clearly curve around the flexure, continuing into the hypothalamus and supraoptic commissures. The peduncular tract diverges dorsalward at a right angle [f.lat.t.v.(10)]. The midbrain is clearly separated from the diencephalon by the posterior commissure (com.post.). In front of that Herrick identified a pretectal region tagged as “pars intermedia diencephali (p.i.d.),” not a standard component of his diencephalic system; next come a pars dorsalis thalami, middle part (p.d.th.m; this is probably just a “middle” part because the “p.i.d.” was considered a caudal part of the same, and either the “ventral habenula” or the “eminentia thalami” was a rostral part) and a pars ventralis thalami (p.v.th). In the prosomeric model the latter must be complemented with the eminentia thalami (em.th.), which we now know belongs to the dorsal alar VTh. These alar territories converge orthogonally ventralwards onto the basal tegmentum full of longitudinal fibers and the cephalic flexure, and also point in the contrary direction toward the habenular region and the roof plate. The arrangement of all elements agrees perfectly with Kappers' schema in **(A)**.

Forebrain neuroanatomic terms used widely during the last 100 years are typically adapted to the *columnar model* of the forebrain, which was first proposed by Herrick (1910) in amphibia and reptilia (review in Herrick, 1948), and was later extrapolated to amniote and several anamniote vertebrates by Kuhlenbeck in the twenties, thirties and beyond (review in Kuhlenbeck, 1973). Many other authors also contributed to this development, particularly with work on diverse mammals, converting this model in the predominant neuroanatomic paradigm until its recent decline. Indeed, the advent of brain molecular marker results accruing since the 1980s has increasingly elicited a concern about the lack of explanatory value and scarce present utility of the columnar model. The change is due in essence to the increasing need to have meaningful morphologic interpretations of gene expression patterns and functions in the brain. The columnar model has revealed itself unwieldy and generally unsatisfactory for aiding the spatially-oriented understanding of observed genoarchitectonic patterns, as well as for extracting causal interpretations of experimental developmental results and transgenic mutant phenotypes (Figures 1A,B, 2–6).

The literature since 1990 shows practically no example of straightforward application of the columnar model to gene expression or mutant phenotype analysis, and the few instances are considered difficult to understand (e.g., Alvarez-Bolado et al., 1995). It has been less obvious that the capacity of the columnar model to inspire insight on brain functions has also reached a low ebb. This capacity seemed high initially, but it gradually was realized that it stood on a simplistic basis, i.e., Herrick (1910) objective to explain forebrain functions as an extension of brainstem columnar functions related to visceral and somatic cranial nerve components. This scenario has led to the substitution of the aged columnar model by more powerful segmental brain models. The latter are historically older (see Orr, 1887; McClure, 1890; Locy, 1895; von Kupffer, 1906; Ziehen, 1906), but had practically been relegated to oblivion under the influence of the dominant columnar model. The modern version of such segmental (neuromeric) models is the *prosomeric model* (Figure 1B; Puelles and Rubenstein, 1993, 2003, 2015; Rubenstein et al., 1994; Puelles, 2013), which embodies a corrected and expanded version of the earlier neuromeric model of Palmgren (1921) and Rendahl (1924). This model's name derives from *prosomeres*, understood as neuromeric developmental units of the prosencephalon or forebrain (irrespective that the model also deals with rhombomeres in the hindbrain; note the prosomeric forebrain also includes the midbrain, whose prosomeres are also called “mesomeres”).

The theoretic underpinnings of forebrain neuromorphology became molecular during the last 40 years, and in so doing registered a readjustment which fundamentally rests on a different axis concept and the role played by neuromeres transverse to that axis (Figures 2A,B). This implied a significant paradigm change in brain neuroanatomy that is still being assimilated as new generations of neuroscientists enter the field. The new paradigm is already prevalent in the subfields of developmental and evolutionary/comparative neuromorphology (Puelles et al., 2013, 2018; Nieuwenhuys and Puelles, 2016). Colleagues that do not follow closely the developmental advances accrued in this field may not see yet the reasons why this change to the prosomeric model is convenient and necessary.

A number of columnar neuroanatomic *terms* unfortunately need to be adapted to the logic of the prosomeric model, in order to obtain full fruits of its heuristic potency. Side-by-side comparison of the columnar and prosomeric models shows roughly a 90° difference in the definition of the brain axis in the rostral forebrain, as well as sizeable differences in the rostral and caudal delimitation of the midbrain (Figures 1A,B; Puelles and Rubenstein, 2015). Fundamental regions of the forebrain such as midbrain, diencephalon (including pretectum, thalamus and prethalamus), and hypothalamus have now subtly different prosomeric definitions. Therefore, I am not writing about whimsical altering of terminology here or there. We deal with a major paradigmatic change in the whole of neuromorphology produced thanks to the evidence of hundreds of gene markers and a mass of experimental results accrued during the last 40 years. We obviously must argue against the traditional terminological conservativeness of neuroanatomists, but, given that scientists will continue to communicate with each other using words, the consequent adjustments will be accepted sooner or later, as happened with important name changes accepted in the past. For instance, the term “hypothalamus” was a neologism as recently as 1893 (His, 1893), substituting the earlier name of “subthalamus” (Forel, 1877).

It is clear that many forebrain anatomic descriptors (e.g., dorsal, ventral, rostral, caudal, anterior, posterior) need to be adjusted to the different axial reference (Figures 1A,B), and some well-known neural structures must be ascribed to natural regions of the brain different than those assumed classically (e.g., the subthalamic nucleus, is a retromamillary derivative found in the retrotuberal basal hypothalamus). One can translate mentally to some extent the new morphologic meaning of the anatomic entities. However, the newer generations will surely prefer more direct and pragmatic general solutions, and I leave aside the important fact that we absolutely will need such solutions in any computerized *ontologies*, since databases are not able to translate mentally. We do not want databases to fix forever the meanings of descriptors, or how we call items in the brain, since terminologies imply theories, hypotheses and assumptions, and these at least will surely change. I believe terminological adaptation to the present paradigm change will emerge gradually, at its own pace, driven by the inevitable semantic needs resulting from continued scientific activity. Old vitiated terms will be found increasingly confusing due to their false implications or assumptions, and will be gradually left aside, to the benefit of more exact alternative terms, wherever they come from. Accordingly, it would be premature at the present time to pretend to offer a fully developed system of solutions to this complex problem (Puelles L. et al., 2012a commented on changes needed for the future hypothalamus concept, whose proposal seems presently impossible; likewise, Puelles, 2016 covered the new midbrain concept, and also proposed some urgent related terminology changes; the present essay will be partly based on these accounts). Probably a diversity of conceivable alternative terms will emerge as more authors start attending to this issue. More and more colleagues will discover that they are being short-changed into confused ideas by the old terminology and/or model. Irrespective that we probably will suffer a transitional chaotic period in semantics (see a remarkable example in Xie and Dorsky, 2017 on the hypothalamus, where both inconciliable columnar and prosomeric models are used at cross-purposes), the new proposals surely will be amply discussed for cogency and usefulness. Eventually, at some point in the future, a new forebrain neuroanatomic nomenclature agreeing or not with the prosomeric model will be convened upon by an international congregation of experts.

The present essay aims to explore in a preliminary way this scenario, first presenting some of the criticisms addressed nowadays to the columnar length axis, which underpin in my opinion the cited paradigm change (Figures 1A,B), and then commenting on the nature of the problems raised at each major forebrain region. Selected examples of potentially changeable terms will be discussed. It will be seen that some aspects of neuroanatomic terminology are changing already, or were changed tentatively in recent times, in order to adapt to the new neuromorphological thinking made possible by the prosomeric model (more on this rationale in Puelles E. et al., 2012a; Puelles et al., 2012b, 2013; Puelles L. et al., 2012a; Puelles, 2013; Puelles and Rubenstein, 2015; Nieuwenhuys and Puelles, 2016).

## Problems With the Columnar Forebrain Axis and the Definition of Longitudinal Columns in the Forebrain

In proposing his columnar model Herrick (1910) contradicted widely accepted ideas on the forebrain length axis which had been systematized shortly before by Orr (1887); His (1893, 1895, 1904); Ziehen (1906), and Johnston (1906, 1909). Herrick postulated that the length axis of the brain (and its landmark, the sulcus limitans of His, dividing alar and basal longitudinal zones) might end in the telencephalon, rather than in the preoptic recess, as the earlier authors had uniformly assumed (Figure 1A; compare Figures 2A, 4, 6). The diencephalon of Herrick was thus a full transverse sector of the neural tube intercalated between the telencephalon, rostrally, and the midbrain, caudally, and included ventrally the hypothalamus (M, Di, Tel, HTh; Figure 1A, see also Figure 3). Herrick's (1910) main interest lay in defining a *dorsoventral* subdivision of the diencephalon into four *longitudinal columns* (*epi*thalamus [ETh], *dorsal* thalamus [DTh], *ventral* thalamus [VTh], and *hypo*thalamus [HTh]; Figure 1A). The words in cursive in the previous sentence correspond to descriptors whose morphologic meaning within columnar interpretation applies the columnar axis concept. The referred forebrain domains do not have the same topologic meaning in the prosomeric model (Figure 1B). The columnar axis was in any case a theoretic construct, because it was not morphologically visible in terms of landmarks, and, moreover, its assumed straightness was contradicted sharply by the cephalic flexure (Figures 2, 3). In Herrick's subsequent work, and that of many of his followers, the abandonment of His's alar-basal axial sulcal landmark led to parallel underplaying of the important alar-basal histogenetic difference in the diencephalic wall. This is precisely one aspect of reality that genes—particularly *Shh* expressed throughout the forebrain basal plate and various other Shh-related genes (Figures 5A–C)—have modernly corroborated, reinforcing our present prosomeric belief that Herrick's “longitudinal columns” actually are *transversal* entities (Figures 4–6).

Another relevant point we have learned with the genes in hand is that true regional boundaries of brain progenitor domains do not habitually coincide with *ventricular sulci*, much used in standard columnar studies for delimitation. Some of the primary molecular boundaries coincide rather with *ventricular ridges* at early developmental stages, notably those adopting a *transversal interneuromeric topology* [e.g., *Shh*-positive ZLI (zona limitans intrathalamica), pretectal *Pax3* and thalamic *Gbx2*; Figures 5A,B, 10; see Lakke et al. (1988), a scanning electron microscopic analysis in the diencephalon]. In any case, both sulci and ridges of the ventricular surface are understood now as tertiary epiphenomena of the morphogenetic histogenetic differences established first by primary molecular boundaries. Moreover, it is very doubtful that genes can code for a sulcus or a ridge, and, even if they could, mechanistic effects merely *shaping* the ventricular surface do not seem efficient characters for evolutionary selection.

The arbitrary columnar concept of what was “longitudinal” in the diencephalon also caused unexplained “impossible” topologic relationships of the “columns” with the roof and floor plates (Figures 1A, 2C, 4, 6A,B), which induced followers of the model to disregard the bending of the brain axis at the cephalic flexure, a constant feature of all vertebrate brains (Figure 3). Some ulterior versions of the columnar model did admit the cephalic flexure and part of the sulcus limitans of His (e.g., Kuhlenbeck, 1973; Altman and Bayer, 1988, 1995; Swanson, 2012; concept represented in Figure 1A), but inconsistently maintained the belief that diencephalic columns were longitudinal.

As regards the theoretically straight length axis of Herrick (1910), it was rarely discussed that there is very poor developmental support for its telencephalic ending. Modern molecular embryology highlights instead the relevant axial causal role of the notochord in establishing the *neural floor plate*, which in its turn induces in antagonistic interaction with roof plate morphogens the *basal plate* and the *alar-basal boundary* or sulcus limitans (see Puelles L. et al., 2012a; Figures 2B, 5A–C). Note the notochord (and accordingly the floor plate) ends rostrally under the mamillary hypothalamic pouch (Ma; Figures 1A,B, 6A; additional molecular evidence in Puelles L. et al., 2012a; Puelles and Rubenstein, 2015). There is no analogous causal underpinning for the postulated columnar brain axis extending hypothetically into the telencephalon; compare (Figures 1A,B). Swanson (2012, 2018) holds speculatively that the *columnar* basal hypothalamus extending into the “basal telencephalon” is induced by the prechordal plate, even though the prechordal plate material does not reach beyond the preoptic region. Moreover, lack of prechordal signaling only causes holoprosencephaly (repatterning and cyclopy), but not a loss of the telencephalon and hypothalamus.

Another point hardly discussed in columnar literature is why the VTh, DTh and ETh “columns,” supposed to be mutually parallel, seem to end “rostrally” at the diencephalic *roof plate*, the major *dorsal* landmark, rather than having a straightforward telencephalic ending, as one would expect. Theoretically, only the ETh should participate in the roof plate, but it is clear that ETh, DTh and VTh reach that longitudinal zone (see Figures 1A, 2C, 4, 6). This conundrum implies that the limiting thalamic “longitudinal” sulci that were used to define these columns somehow are less longitudinal than was assumed, being in fact disposed obliquely, or even *orthogonally*, to the roof plate. The same inconsistent conundrum emerges again in the opposite direction for DTh and VTh. The theoretically “caudal” end of these columns meets *orthogonally* the longitudinal basal plate (Figures 1A, 2C, 4, 6). This again should be impossible if DTh and VTh are longitudinal structural entities. It suggests they are in fact transversal domains, as was thought by major contemporaries (Kappers, 1947; Figure 6A). According to this morphologic consistency analysis, something seemed to be wrong with the columnar forebrain axis and the conclusion that the diencephalon contains four “longitudinal” columns.

The *prosomeric model* uses as axial reference the molecularly-defined floor plate and alar-basal boundary (primary patterns, as opposed to tertiary phenomena such as ventricular sulci used by Herrick, 1910). The modern alar-basal boundary only differs from the sulcus limitans of His in ending under the optic chiasma rather than above it (Figures 1B, 2B, 3, 5A–C, 10; Puelles L. et al., 2012a; Paxinos and Franklin, 2013; Puelles and Rubenstein, 2015); note it is theoretically advantageous to have the eyes and chiasma as alar structures; otherwise you have a sensory pathway entering the basal plate, as happens undiscussed in the columnar view (e.g., Swanson, 2012, 2018). Our model resolves all the mentioned columnar conundrums, revealing that VTh, DTh and pretectum are alar subregions of straightforward transversal neuromeric units of the diencephalon (p1–p3; Figures 1B, 5, 10); note particularly how the observed topologic relationships with the roof, basal and floor plates are resolved. The diencephalon accordingly lies altogether caudal to the hypothalamus, and the basal plate does not extend into the telencephalon (Figures 2B, 5B,C, 10).

The inescapable morphologic problem of the columnar model, which causes a host of secondary problems, is that the forebrain axis was arbitrarily formulated, and turns out to be inconsistent with modernly investigated causal mechanisms, as well as with many molecular and structural patterns of the forebrain wall.

## Midbrain Terminological Problems

**Figure 7:**
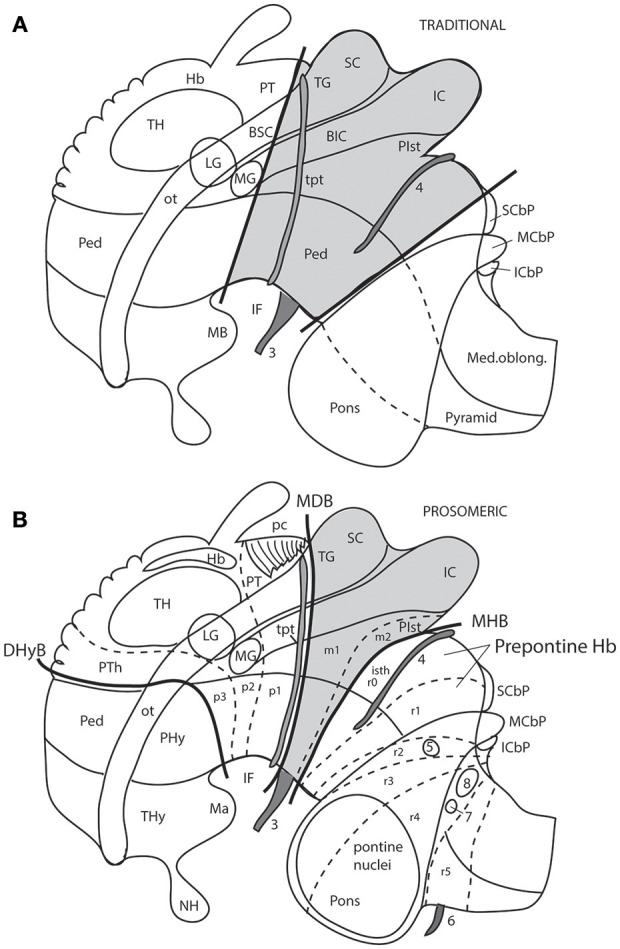
Schemata from Puelles (2016) illustrating the different columnar and prosomeric models of the midbrain, in the context of neighboring diencephalic and hindbrain areas (no copyright permission required). Several superficial tracts were added for anatomic reference: the optic tract (ot), the cerebral peduncle and related medullary pyramid (Ped), the brachia of the superior and inferior colliculi (BSC, BIC), the transverse peduncular tract (tpt, related to the basal optic root), the extraneural oculomotor and trochlear cranial nerve roots (3, 4), and the middle (pontine) cerebellar peduncle (MCbP). **(A)** This drawing illustrates the conventional classic midbrain concept one finds in most textbooks, still abundantly used by clinicians. Thick straight black lines roughly indicate the rostral and caudal midbrain borders (the rostral limit being that suggested tentatively by His, 1893). It passes rostrally to the transverse peduncular tract (tpt; compare the position of this landmark in the caudal pretectum in **B**). It also ascribes most of the interpeduncular fossa (IF) to the midbrain, when in fact this depression is largely diencephalic and rhombencephalic (compare in **B**). The caudal midbrain boundary abuts the upper limit of the MCbP (see also the superior and inferior cerebellar peduncles; SCbP, ICbP; all of them in rhombomere 1, i.e., caudal to the isthmic segment). **(B)** This schema shows the more precise prosomeric concept relative to the same anatomic landmarks. I added with thin dash lines the borders between the diencephalic prosomeres (p1–p3) and the posterior commissure landmark (pc), important for placing the rostral mes-diencephalic boundary (MDB); this landmark jointly with the subcommissural organ characteristically mark the whole dorsal extent of the pretectum, though the crossed fibers converge laterally into a caudal subregion, the “commissural pretectum.” The *transverse peduncular tract* carrying retinal fibers of the basal optic root ascends peripherally to the peduncle just in front of the pretecto-mesencephalic border or MDB. This drawing does not depict the tectal gray formation that lies within the midbrain rostrally to the superior colliculus (compare TG in Figure 1). Caudally to the inferior colliculus there is the m2 segment, representing the preisthmus region. Only a small portion of the interpeduncular fossa (IF), coinciding with the oculomotor nerve root, corresponds to the midbrain. The midbrain-hindbrain boundary (MHB) separates m2 from the isthmus (isth = r0). Part of the literature confusingly mixes the isthmus with the r1 proper under the name “r1,” using the rationale that the isthmus is not a proper neuromere; the contrary was held by His (1893), Palmgren (1921), and Vaage (1969, 1973), and strong molecular evidence was presented recently by Watson et al. (2017); once the need to separate the isthmus as an additional hindbrain neuromere was heeded, it seemed best to call it r0, rather than change the numbers of all other rhombomeres. The r0 and r1 neuromeres jointly form the prepontine hindbrain (compare with **A**), which lies under the range of effects of the isthmic organizer, thus sharing some features, including dorsal raphe, cerebellar, mesV, and interpeduncular structures, regardless of their respective differential identities. Several hindbrain cranial nerve roots were added to **(B)** in order to see their topography relative to specific rhombomeres (indeed, the nerve roots are valid landmarks to access relative rhombomeric position in all vertebrates): trigeminal root in r2 (5), facial and vestibulo-cochlear nerves in r4 (7, 8), and abducens root in r5 (6). Note the basilar pontine nuclei occupy exclusively the ventral region of r3 and r4. Nevertheless, r2 is also ascribed to the pontine region, because it contains massive fiber bundles of the pontine MCbP coursing into r1 rostrally to the trigeminal root in r2. Since the cerebellum is formed in r0 and r1, all peduncles need to reach these segments in order to find entrance into the cerebellum. This was not appreciated in older times. The thick black line crossing the optic tract in front of the prethalamus (PTh) and behind the peduncular hypothalamus (PHy) is the diencephalo-hypothalamic boundary (DHyB). The thalamus is symbolized by an ovoid mass plus the lateral and medial geniculate bodies (LG, MG). Note the MG represents topologically the ventralmost thalamic mass, actually lying *ventral* to the LG. Both LG and MG lie close to the interthalamic limit (PTh/TH).

The caudal midbrain limit was traced classically along the “ponto-mesencephalic sulcus” that runs just above the pons (Figure 7A). The rostral midbrain limit, or mes-diencephalic border, was given classically by an imaginary plane passing in front of the superior colliculus (normally across the posterior commissure; Figure 7A). This limit extended under the medial geniculate body and ended ventrally at the upper end of the interventricular fossa, close to the mamillary bodies (Figure 7A). This boundary was proposed by His (1893); Figure 2A) who acknowledged it was tentative and arbitrary, due to lack of suitable landmarks (he did not recognize the posterior commissure as the relevant landmark he needed). However, his tentative border became a dogma for over 100 years. Curiously, His (1893) also proposed an essentially correct isthmo-mesencephalic *caudal* boundary of the midbrain in the same work (Figure 2A), but this was not accepted by conventional neuroanatomy. As a consequence of these long-standing midbrain limits, the whole interpeduncular fossa and visible *pes pedunculi*, plus the lemniscal trigone and the caudal pretectum, were held to be mesencephalic, and so were both oculomotor and trochlear nerves (Figures 1A, 7A).

**Figure 8:**
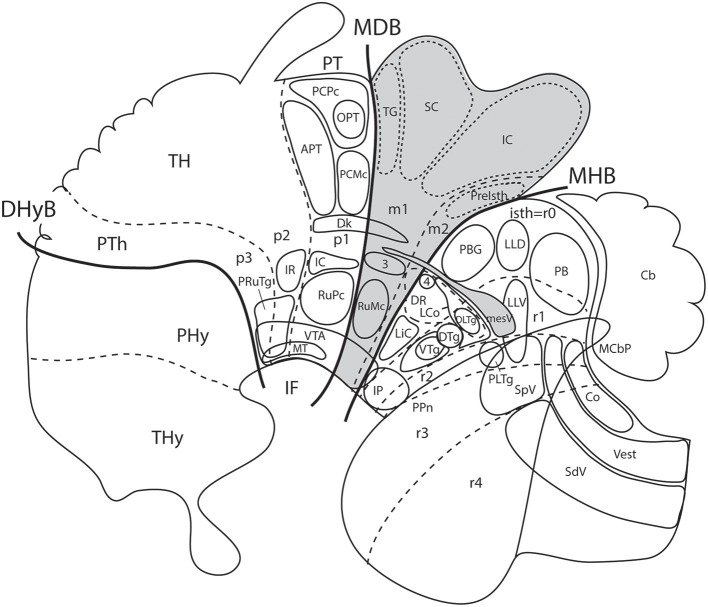
Schema from Puelles (2016) (modified from those in Figure 7; no copyright permission required) showing truly mesencephalic centers (gray background) according to the prosomeric model, as opposed to neighboring diencephalic or rhombencephalic (prepontine) formations that have been implicated in erroneous ascription to the midbrain within classic neuroanatomic usage inspired in the columnar model. For specific ascriptions to the isthmic neuromere (r0), see Watson et al. (2017). The trigeminal mesencephalic nucleus is thought to originate in the midbrain and partly migrate into the prepontine hindbrain along the mesV tract. The ventral nucleus of the lateral lemniscus (LLV) has been shown to originate in r4, and migrates subsequently along the tract into its final position (Di Bonito et al., 2013, 2017). The abbreviations correspond to standard ones used in recent rodent atlases. MDB, mes-diencephalic boundary; MHB, midbrain-hindbrain boundary.

This classic concept of the midbrain limits has not stood the test of molecular data. Gene expression patterns and experimental embryology data (fate mapping and repatterning studies; studies on secondary organizers) have concluded decisively that both traditional limits defined above are inexact, and even causally impossible, because of regulatory antagonistic developmental mechanisms that do not allow truly diencephalic or hindbrain domains to *be “mesencephalic”* in molecular profile and fate, or viceversa (e.g., rotation experiments of Marín and Puelles, 1994; a prospective pretectal nucleus cannot develop such fate if placed inside the midbrain field). It has been shown, moreover, that the old “midbrain” (Figure 7A) does not represent a developmental unit, because it is too inclusive: it arbitrarily encompasses diencephalic derivatives rostrally and hindbrain derivatives caudally (Figures 1B, 7B, 8). The new, more restricted concept of the midbrain is consistent with gene patterns, causal mechanisms (e.g., effects of the isthmic organizer), and modern notions about neuromeric structure of the neural tube (the prosomeric model).

**Figure 9:**
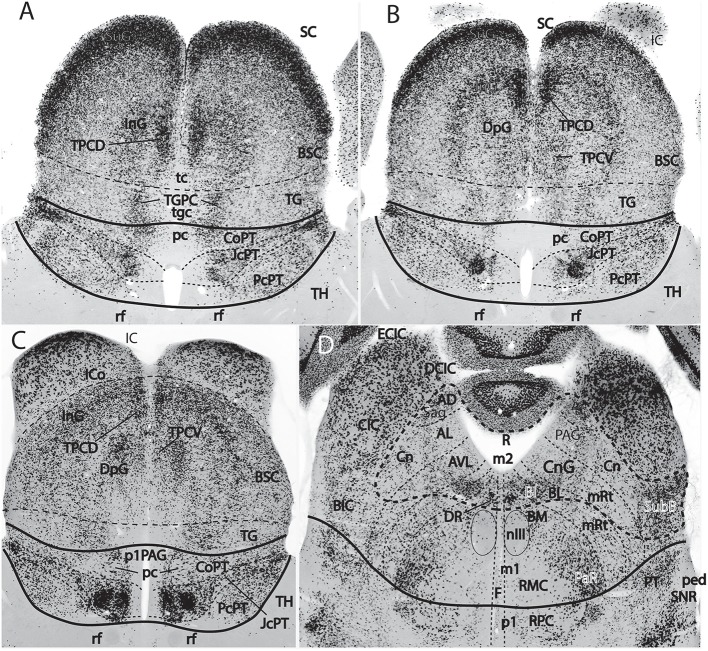
Figure extracted from Puelles E. et al. (2012a) showing fundamental pretectal and midbrain structure in four horizontal sections **(A–D)** in dorsoventral order, illustrating in particular the adult mouse thalamo-pretectal and pretecto-mesencephalic boundaries (thick black lines; no copyright permission required). This material is *in situ* reacted for Gad67, thus showing selectively midbrain alar GABAergic neurons in superficial, intermediate and deep periaqueductal strata (note there are practically none within the neighboring thalamus). The pretecto-mesencephalic border passes just behind the posterior commissure and in front of the distinct superficial, layered and retinorecipient formation identified as tectal gray (pc; TG; **A–C**). The TG differs from the superior colliculus (SC) in the number of superficial GABAergic neurons, as well as in the aspect of its periaqueductal formation. The novel dorsomedial tectal elements termed dorsal and ventral tectal paracommissural (longitudinal) nuclei are shown in position (TPCD, TPCV; **A–C**); there is also a similar tectal gray paracommissural nucleus (TGPC; **A**). The pretectum appears divided into precommissural (PcPT), juxtacommissural (JcPT), and commissural (CoPT) anteroposterior domains with differential structure and molecular profile (**A–C**; Ferran et al., 2008, 2009). The inferior colliculus (IC) starts to appear in **(B**,**C)**, but is shown fully in **(D)**. The section in **(D)** is slightly oblique from left to right, so that the right side passes somewhat under the IC, showing slightly more of the caudally underlying preisthmus or m2-derived midbrain territory (formations enclosed by the thick dash line; note the relevant subpial subbrachial nucleus, SubB, at the right). The small dash lines in **(D)** refer to the limits between different dorsoventral midbrain sectors visible at this level. Neither the oculomotor nucleus (nIII) nor the magnocellular nucleus ruber (RMC) in the basal plate contain GABAergic neurons, but the latter is surrounded laterally by a distinct mass of such cells, forming the pararubral nucleus [PaR; **(D)**; this population derives from the parabasal *Nkx2.2*-positive band illustrated in chicken in Figure 5C, and expresses this marker in the adult]. GABAergic cells are also present as a subpopulation in the parvocellular nucleus ruber lying in the pretectal tegmentum, also partly surrounded by the PaR (RPC, **D**).

The first precise definition of the midbrain (which was consistent with His (1893) pioneering formulation of the isthmo-mesencephalic boundary) was proposed by Palmgren (1921), after comparative developmental studies in several vertebrate species, well before the advent of corroborating genetic evidence. Vaage (1969, 1973) provided additional developmental evidence consistent with Palmgren's model in chick embryos. Puelles and Martínez de la Torre (1987), García-Calero et al. (2002), Hidalgo-Sánchez et al. (2005), and Ferran et al. (2007, 2008, 2009) later built upon these precedents, addressing successively the caudal and rostral midbrain boundaries. Additional gene marker evidence was collected by Puelles E. et al. (2012a) for the adult mouse brain. The most relevant markers are the transcription factors *Otx2* (whose forebrain expression domain permanently ends caudally at the caudal midbrain boundary after neurulation) and *Pax6* which marks early on the alar pretecto-tectal limit in all vertebrates (i.e., the rostral midbrain boundary, passing *behind* the posterior commissure; Figures 7B, 8, 9, 13A).

Figure 8 illustrates well-known brain nuclei that were classically thought to be mesencephalic (still so in Swanson, 2012, 2018), which turn out to be either diencephalic or hindbrain derivatives under the modern molecular midbrain definition. The trochlear nucleus and nerve are isthmic (Watson et al., 2010, 2017), while the interpeduncular nucleus complex is isthmic- and r1-derived (Lorente-Cánovas et al., 2012; IP in Figure 1B). The dorsal and ventral tegmental nuclei and the locus coeruleus (Aroca and Puelles, 2005; Aroca et al., 2006) clearly are r1-related. Serotonergic raphe cell populations are rhombencephalic in general, including the dorsal raphe nucleus, which was classically thought to be mesencephalic (Alonso et al., 2012); there is only a small rostrally migrated subpopulation of the dorsal raphe nucleus that finally lies in the caudal midbrain (m2 prosomere; identified as “midbrain DR” by Alonso et al., 2012). The mesencephalic trigeminal nucleus of all non-mammals lies exclusively in the midbrain, while in mammals it also extends caudally into the isthmus and rhombomere 1 (mesV in Figure 8); this evolutionary difference suggests that the mammalian mesV cells probably have midbrain origins and then migrate tangentially into isthmus and r1. Another modern conclusion is that the decussation of the brachium conjunctivum (superior cerebellar peduncle) lies not in the midbrain, but across the isthmic floor (Paxinos and Franklin, 2013; Watson et al., 2017; Martínez-de-la-Torre et al., 2018).

**Figure 10:**
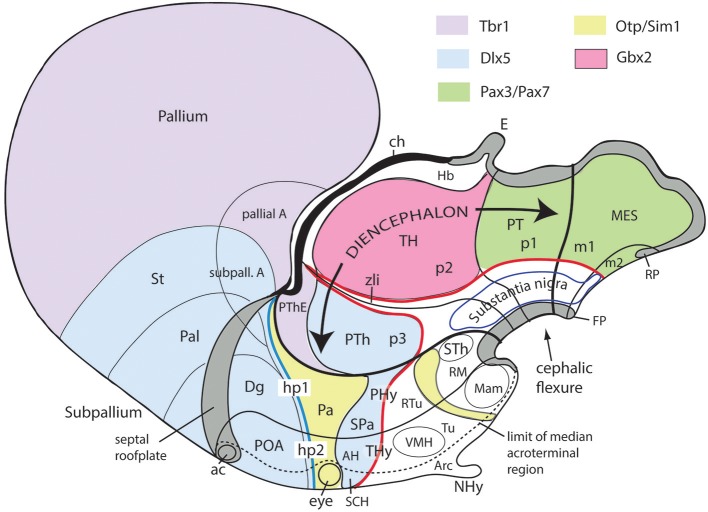
Schematic map copied from Puelles (2015) of various gene expression patterns that agree with prosomeric analysis of the forebrain (largely centered on alar plate domains of the different prosomeres across midbrain, diencephalon and hypothalamus; no copyright permission required); alar-basal limit in red, though the zona limitans transverse spike (zli) is independent from the basal plate, irrespective of its molecular similarity (Shh expression; see Figure 5B). The mesodiencephalic extent of the dopaminergic substantia nigra and associated ventral tegmental area is mapped, as well as the distinct perimamillary/periretromamillary basal hypothalamic band, in yellow (see text); the separate alar hypothalamic paraventricular area (Pa; also in yellow)shares these particular markers (but not others). Dlx5 is shared among given prethalamic (PTh), hypothalamic (SPa, AH, SCH) and subpallial (St, Pal, Dg, POA) domains typically producing GABAergic neurons. A thick blue line represents the hypothalamo-telencephalic boundary; note telencephalic subpallial inclusion of the POA due to shared gene expression, and local ending of the roof plate at the anterior commissure (ac). Hypothalamic alar and basal subdivisions and some individual nuclei mentioned in the text are identified.

The midbrain is divided into unequal mesomeres 1 and 2 (m1, m2; Figures 5A, 7B, 8, 10; Hidalgo-Sánchez et al., 2005; Puelles, 2013); this division was already affirmed, even if not clearly documented, by Palmgren (1921) and Vaage (1969, 1973). However, these authors thought that m2 was an *atrophic* neuromere that produced no neural derivatives (a very odd idea, that discredited the notion for a long time). However, Hidalgo-Sánchez et al. (2005) demonstrated both that a particular molecular profile exists in m2 (within the field of midbrain *Otx2* expression, thus corroborating its midbrain neuromeric status distinct from m1) and showed some clearcut alar and basal m2 derivatives (Figure 9D; see also Puelles E. et al., 2012a). This development led to the modern concept of a distinct midbrain m2-derived domain, also called *preisthmus*, which lies intercalated between the inferior colliculus and the isthmus proper (Figures 7B, 8, 9, 10). The corresponding alar region contains in its intermediate and superficial strata what classically was identified as the cuneiform nucleus or nuclear complex; rodent atlases usually wrongly distribute this complex across both preisthmus and isthmus (Puelles E. et al., 2012a; Puelles, 2016).

The classical “posterior pretectal nucleus” has been modernly recognized to be mesencephalic and renamed “tectal gray,” following previous usage in non-mammalian tetrapods (TG in Figures 1B, 8, 9). The TG is truly mesencephalic, because it lies caudal to the posterior commissure, and it lacks *Pax6* expression typical of neighboring pretectal areas (Ferran et al., 2008).

The midbrain alar plate is thus built by a rostrocaudal sequence of four *major structures*, rather than just the two classic colliculi: *tectal gray, superior colliculus, inferior colliculus* (all three within m1), and *alar preisthmus* within m2 (Figures 1B, 7B, 8–10). As regards the midbrain basal plate, the oculomotor nucleus complex lies within m1, while m2 (preisthmus) is devoid of motoneurons, since the trochlear nucleus is isthmic (Figure 8; Watson et al., 2017). The substantia nigra and ventral tegmental area, which are conventionally ascribed only to the midbrain in the old model (Figure 7A), actually represent in the new scenario a plurineuromeric isthmo-meso-diencephalic complex that extends from the isthmus to the rostralmost diencephalon (Figures 7B, 10; Medina et al., 1994; Puelles and Medina, 1994; Verney et al., 2001; review in Puelles E. et al., 2012a; Puelles et al., 2012b, 2013; Puelles L. et al., 2012a; Puelles, 2013). Modern experts on the development of this complex already use routinely the expression “mesodiencephalic SN/VTA” (see also a comparative review in tetrapods by Marín et al., 1998). Another typical tegmental midbrain element is the red nucleus. However, only the magnocellular red nucleus is mesencephalic, while the parvocellular red nucleus is pretectal diencephalic (RMC, RPC; Figure 9D; Puelles E. et al., 2012a); the classics underlined that the parvocellular red nucleus was limited rostrally by the retroflex tract, and the latter is the transversal landmark that limits thalamus (p2) from pretectum (p1) (see rf in Figures 1A,B, 4).

Other specific points possibly merit detailed examination. For instance, the “midbrain locomotion center” (MLC) is commonly identified anatomically with the cuneiform nucleus, an alar preisthmic derivative which we identify within m2 (Shik and Orlovsky, 1976; Mori et al., 1977; ten Donkelaar, 2011; ten Donkelaar et al., 2018). The literature however tends to conceive the cuneiform nucleus as a tegmental (basal) nucleus, which it is not, if it really is preisthmic (the cuneiform nucleus actually lies just *caudal* to the inferior colliculus, but still in the alar plate). However, the MLC also has been said to lie close to the pedunculopontine tegmental nucleus (PPnTg), which is a well-known cholinergic and NOS positive population which lies within tegmental r1. Therefore, if the MLC is really tegmental in position, then it is incorrectly identified as “cuneiform nucleus,” a structure that is distant from the PPnTg (separated by the whole isthmus). Contrarily, if it really is alar preisthmic, then it has been wrongly identified close to the PPnTg in the r1 tegmentum. Considering the alar/basal difference and that these two sites are separated by the whole isthmus, as well as the standard imprecision of atlases on this brain region, it is possible that the identification of the original physiologic electrode recording sites as being at the “cuneiform nucleus” was inexact. The MLC thus perhaps lies instead within the isthmus, where it may well be a tegmental basal structure to be found next (just rostral) to the PPnTg. Unfortunately, if it is isthmic, or belongs to r1, then it does not merit the given name “midbrain locomotion center.” I hope that present discussion of the midbrain limits helps in resolving this conundrum.

The diverse points made above on the general subject of “midbrain terms” show that most of the problems are conceptual, and relate to the wrong definitions used classically for the rostral and caudal limits of this brain part, or result from poor knowledge of its basic subdivisions m1 and m2. Once the modern molecularly-based (and experimentally corroborated) definition of the relevant boundaries is seen as the natural one (not man-made, as the old one was), it only remains for us to demand better atlases than we have now (e.g., see the already corrected chick brain atlas; Puelles et al., 2007, 2018).

The main new names that have been proposed for the midbrain include “tectal gray” (for the stratified retinorecipient center found just rostral to the superior colliculus, previously wrongly ascribed to pretectum as “posterior pretectal nucleus”), and “preisthmus” (for the adult derivatives of the alar and basal domains of the m2 prosomere, largely unnoticed by the classics). I proposed that the “superficial cuneiform nucleus” term, whose diversified usage has led to substantial confusion in various atlases and in the literature on the midbrain locomotion center, be substituted by the neologism “subbrachial nucleus”, referring to the apparent position of the superficial preisthmus immediately under the brachium of the inferior colliculus [SubB: Figure 9D; Puelles, reference atlases issued in 2009 for the public Allen Developing Mouse Brain Atlas, developingmouse.brain-map.org; Puelles E. et al. (2012a)]; this new term already appears used in some rodent atlases (Watson and Paxinos, 2010; Paxinos and Franklin, 2013; Paxinos and Watson, 2014). Finally, a previously unrecognized dorsal paramedian subzone of the collicular plate has been recently identified as producing *outer (dorsal) and inner (ventral) paracommissural tectal nuclei* (TPCD, TPCV; Figure 9; Puelles E. et al., 2012a); the TPCV was first reported in mammals as a “tectal longitudinal column” (TLC; Saldaña et al., 2007); it includes a rostral portion that surpasses rostrally the superior colliculus and relates instead to the tectal gray, forming actually a “tectal gray paracommissural nucleus,” or TGPC. The related TPCD was mentioned in that publication as a “dorsal column,” which was further characterized by Aparicio and Saldaña (2014), who identified now both nuclei as TLCv and TLCd (the TLCd/TPCD was corroborated as a GABAergic population, as had been shown previously by Puelles E. et al., 2012a; their Figures 10.5–10.8; whereas the TLCv/TPCV is glutamatergic; Aparicio and Saldaña, 2014). The “paracommissural” names I propose derive from our previous independent analysis of an apparent homolog of one of these nuclei in the avian brain (Puelles et al., 2007). The descriptor “longitudinal” proposed by Saldaña and colleagues seems less specific than “paracommissural” regarding positional characterization, and I think there is advantage in explicitly referring to their position close to the tectal gray (tgc), tectal (tc), and intercollicular (icol) median commissures (TGPC; TPCD; tgc, tc; Figure 9A). The connections of the novel TPCV and TPCD nuclei apparently relate them, respectively to the auditory and visual systems (Saldaña et al., 2007; Aparicio and Saldaña, 2014).

## Diencephalon Terminological Problems: General Issues

Figures 1B, 5A, 7A,B, 10 illustrate how the modern prosomeric model deals with the diencephalic forebrain region in contrast to the conventional columnar tradition (Figure 1A). First, the whole pretectum is diencephalic, as redefined by anatomic landmarks (retroflex tract and posterior commissure) and by molecularly stable *Pax6* expression *antagonistic* to the isthmic organizer-controlled midbrain molecular profile (see other pretectal markers in Ferran et al., 2007, 2008). Columnar authors usually ascribed the caudal pretectum to the midbrain and were rather vague about the rest, since in their model it could only enter into the categories of either epithalamus or dorsal thalamus, not being allowed as a distinct diencephalic component because this region was clearly transversal (Figures 1A, 2C, 4, 6B). Secondly, the hypothalamus is no longer held to be diencephalic (whereas it represented the columnar diencephalic floor-plus-basal domain; Figure 1A), due to the prosomeric definition of the forebrain axis as ending within the acroterminal hypothalamic area (Figure 1B; Puelles and Rubenstein, 1993, 2003, 2015; Rubenstein et al., 1994; Puelles L. et al., 2012a; Puelles, 2013; Puelles et al., 2013, 2015; Ferran et al., 2015). The prosomeric hypothalamus is accordingly conceived instead as lying rostral to the diencephalon and forming the rostralmost forebrain region, the *secondary prosencephalon* (basically in agreement with His, 1893, 1895, 1904; Figures 2A,B). This last region encompasses in vertebrates also the eye vesicles and the telencephalon as alar outgrowths. However, the prechordate *Amphioxus* has a molecularly recognizable rostral hypothalamus that lacks eye or telencephalic evaginations (Albuixech-Crespo et al., 2017); this proves that the ancestral forebrain axis ended in the hypothalamus. The left side hypothalamus (alar and basal) is continuous with the right side hypothalamus across the rostromedian *acroterminal area* (Figure 10; neologism introduced by Puelles L. et al., 2012a; Ferran et al., 2015; Puelles and Rubenstein, 2015). The shared alar-basal boundary of the whole forebrain distinctly separates (after use of early molecular markers, neurogenetic labeling, or differentiation markers; Figures 2B, 5) continuous alar and basal zones across the midbrain, diencephalon and hypothalamus (roughly as defined by His, 1893, 1895, 1904; Figure 2A). In the prosomeric model, the telencephalon and eyes are singular *alar* hypothalamic derivatives that evaginate and show differential growth and patterning (Figures 1B, 10). The “hypothalamus” can be conceived more correctly as a “hypotelencephalon,” *sensu topologico stricto*. His (1893) proposed this prefix –“hypo-” = Greek synonim of “under” or “sub”- because he held the hypothalamus (like its antecedent, the subthalamus) to be an exclusively basal domain, whereas the thalamus proper was alar (Figure 2A); it thus made sense to name one domain as lying “under” the other, but this sense is different (about 90°) from that used later in the columnar model (Figure 1A). It eventually was realized using the genoarchitectonic perspective that both diencephalon and hypothalamus have basal and alar parts, and one lies caudal to the other (Figures 1B, 5, 10). One minor terminological problem that arises at this point is that the name “third ventricle” was traditionally applied to the old larger diencephalon inclusive of the hypothalamus. We now need to distinguish rostrocaudally distinct *hypothalamic and diencephalic parts of the third ventricle* (it seems not advisable to alter the number of ventricular cavities).

The “*diencephalon proper*,” an expression we have often used remembering the *diencephalo sensu stricto* of His (His, 1893, 1895, 1904), refers colloquially to the smaller prosomeric diencephalon. This lies intercalated anteroposteriorly between the secondary prosencephalon and the redefined midbrain. It represents a sizeable complete tubular sector of the neural tube which possesses bilaterally all four major longitudinal zones: floor, basal, alar and roof plates (Figure 10). Note the columnar model defined the hypothalamus as the basal and floor domain of the traditional diencephalon; as a consequence of the different axis used, the true basal and floor diencephalic domains of the prosomeric diencephalon proper are very differently placed—e.g., caudal to the mamillary and retromamillary regions-; these regions were substituted in columnar interpretations by the somewhat interlocked concepts of “prerubral tegmentum” and “posterior hypothalamus,” which allowed an *ad hoc* and theoretically inconsistent continuity between basal hypothalamus and basal midbrain (inconsistent because this bridge is visibly orthogonal to the postulated “longitudinal” axis of the columnar model; see Figures 1A, 3). Due to its complete dorsoventral structure, the prosomeric diencephalon proper resolves satisfactorily the observable relationships of its neuromeric subdivisions with the roof and floor domains (Figures 1B, 5A, 10). Significantly, it limits rostrally with the whole secondary prosencephalon, i.e., both with the hypothalamus and the telencephalon (Figures 1B, 10). It should be known that a variable rostrodorsal alar portion of the prethalamic diencephalon evaginates jointly with the telencephalic vesicle, entering into its definitive medial wall, and causing some anatomic peculiarities at this largely hidden area (Figure 1B; see below, as well as Lakke et al., 1988, their Figures 4, 5A, which are consistent with our Figure 4).

The diencephalon is divided into three diencephalic prosomeres (p1–p3, Figure 1B; always numbered in caudo-rostral order). These were first clearly recognized in birds, reptiles and mammals by Rendahl (1924). He identified them as synencephalon (p1), posterior parencephalon (p2), and anterior parencephalon (p3), terms still found occasionally in the literature (e.g., in Puelles and Martínez de la Torre, 1987, or in Lakke et al., 1988, cited above). Rendahl ascribed the hypothalamus to p3, perhaps in partial abeyance to Herrick's (1910) model; this inconsistency was already corrected by Puelles and Martínez de la Torre (1987) (review with schematics in Puelles, 2018). On formulating the prosomeric model (Puelles and Rubenstein, 1993; Rubenstein et al., 1994), we preferred to give terminological protagonism to the much more common terms “pretectum” (p1), “thalamus” (p2), and “prethalamus” (p3), which we redefined in agreement with the novel molecular evidence, but in substantial topologic agreement with a good number of classic observations regardless of the offered non-neuromeric interpretations [e.g., Bailey, 1916; (Figure 4); Miura, 1933; Gilbert, 1935; Herrick, 1936 (Figure 6B), Coggeshall, 1964; Altman and Bayer, 1988]; indeed, the embryos show transversal ventricular ridges rather than longitudinal ventricular sulci as mutual boundaries of these diencephalic domains (Figures 5A, 6A; see the scanning electron microscopic study of Lakke et al., 1988); the implied necessary error lies in the arbitrary columnar axis.

All true pretectal nuclei are diencephalic, building the molecularly distinct alar plate of the p1 diencephalic prosomere (Ferran et al., 2007, 2008; Puelles E. et al., 2012a; Figures 1B, 5A, 7B, 8–10); this means that a pretectal molecular character, as explored by Ferran and collaborators, excludes being “thalamic” or “epithalamic,” as well as being “mesencephalic.” The “thalamus” and “prethalamus” terms substitute for the outdated columnar ones “dorsal thalamus” and “ventral thalamus,” respectively, emphasizing with the new prefix that their mutual topologic relationship is strictly *anteroposterior* (“pre-” used in thalamus/prethalamus exactly as we already used before tectum/pretectum; Figures 1B, 5, 7, 8). Note also that in the prosomeric model (Figure 1B) the epithalamus or habenular region is no longer a fundamental component of the diencephalon, being listed merely as a distinct hyperdorsal subregion of the thalamus (alar p2; ETh; Hb; hab; hb; Figures 1B, 7, 10, 13B), found next to the local roof plate, which displays the unique pineal gland (ep/E; Figures 1, 5A,B, 10). Another distinct hyperdorsal subregion characterizes the prethalamus (p3), and is termed by us the “*prethalamic eminence*” (PThE/pthe). The latter was known in classic works as the “thalamic eminence,” because various authors did not distinguish in adults the respective ventral/dorsal thalamic derivatives and perhaps wrongly thought this eminence represented the rostral pole of the whole thalamic mass (however, Gilbert, 1935 used that term knowing the structure was ventral thalamic). However, for molecular and topologic reasons it is now very clear that the hyperdorsal diencephalic subregion that builds an eminence at the back of the interventricular foramen (next to the local roof chorioidal tissue) lies rostral to the thalamo/prethalamic limit, the zona limitans intrathalamica (PThE/pthe; zli; Figures 4, 5, 10, 13A); accordingly, it must be ascribed to the prethalamus (PTh; Figures 1B, 5; alar p3), and named accordingly “prethalamic eminence” (as many recent authors are already doing). The stria medullaris tract runs *longitudinally* through the PThE before reaching the habenular region. This was thought to be a *ventrodorsal* course in columnar accounts, but posed another semantic conundrum, because the tract's position parallel to the thalamic chorioidal taenia (Figure 1A), an obvious *longitudinal* roof plate landmark, remained unexplained these last 100 years. Similarly, Swanson (2012) has a schema where the whole chorioidal fissure, a straightforward roof plate derivative (Figures 1A,B), is figured as a ventrodorsally oriented component of the early embryonic lateral forebrain wall.

**Figure 11:**
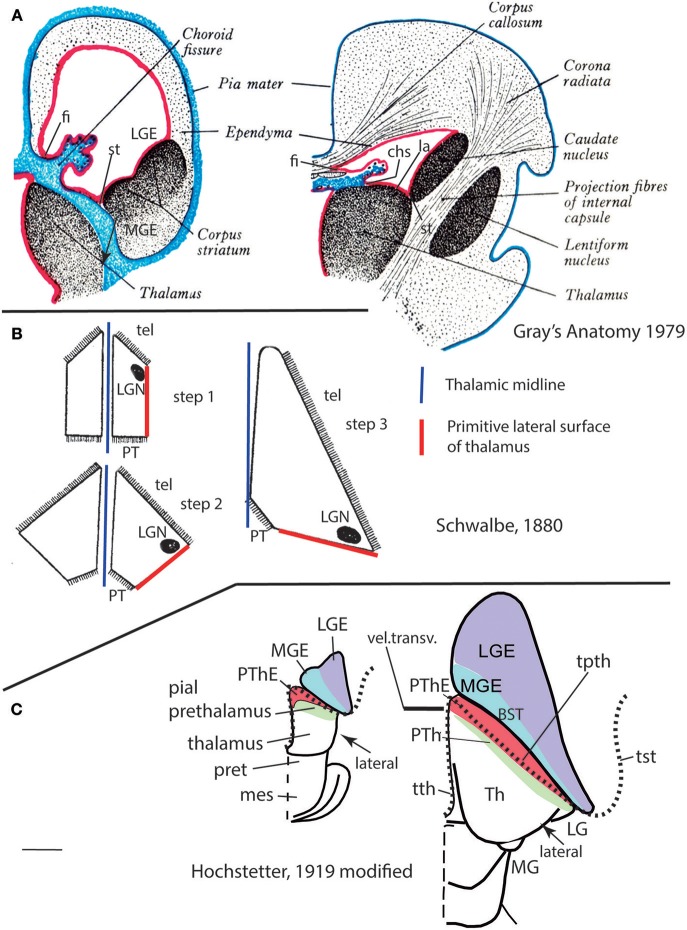
Schematics taken from the literature (all partly modified), to illustrate the classic concept of the lamina affixa **(A)**, the morphogenetic deformation of the thalamus **(B)** and my new emphasis on the associated deformations at the prethalamus and prethalamic eminence, bearing on a new conception of the taenial insertions of the chorioidal fissure **(C)** (no copyright permission required). **(A)** Was extracted from the 1979 British edition of Gray's Anatomy. It illustrates precisely how many classic authors imagined a partial adhesion called “lamina affixa” occurred between overlapping parts of the thalamus and the medial wall of the telencephalon (see an equivalent schema in Dèjerine, 1895). A medial part of the hemispheric wall jumping between the hippocampal fimbria (fi) and the stria terminalis locus (st) was supposed to be primarily chorioidal in texture (leaving unexplained how such tissue derives from the roof plate). Only a dorsal part of it, called “pars libera,” was held to contribute to the development of the definitive adult chorioidal fissure (marked as chorioid fissure at left). However, a lower part of the initial chorioid tissue was imagined to adhere to the thalamus, forming the “lamina affixa” (la; at right). The figured classic conception is conjectural, since it holds without demonstration that one half of the primary medial telencephalic wall adheres to upper and lower parts of the thalamic pial surface. The chorioid-thalamus adhesion (la) would obscure the original chorioidal taenia *imagined* as inserted primarily next to the prospective stria terminalis (st), creating an apparently novel “thalamic taenia” of the chorioidal fissure at the so-called chorioidal sulcus of the thalamus (chs; right side; note there is already a thalamic taenia associated to the roof of the 3rd ventricle). The stria terminalis site was originally held to be associated to the “corpus striatum” [meaning the whole mass of basal ganglia; we now know the st is associated specifically to the lateral ganglionic eminence (LGE; left side), whereas the pallidum and diagonal formations, including the periventricular bed nuclei of the stria terminalis, are derivatives of the medial ganglionic eminence (MGE; left side); see Puelles et al., 2013, 2016]. After the conjectured fusion, the part of the upper thalamic surface covered by the chorioid lamina affixa would apparently protrude at the floor of the lateral ventricle, forming with the medial ganglionic eminence the “terminal” or “opto-striatal” sulcus, a.k.a. as “thalamo-striatal” sulcus. Another imagined adhesion process with consequences was that of the primary lateral thalamic pial surface with the “corpus striatum,” which would allow the thalamic fibers to reach the internal capsule (right side of **A**; this second conjecture was supported expressly by Dèjerine (1895), but is no longer widely supported presently, since the internal capsule fibers are now known to course first internally through the prethalamus—crossing the reticular nucleus—and only afterwards access the telencephalic stalk through the alar peduncular hypothalamus—e.g., see Puelles and Rubenstein (2003, 2015); see also Figure 10; remarkably, Swanson (2012) still postulates implicitly in his rat flat map that thalamic fibers reach the telencephalon across a pial adhesion, and not through the prethalamus). **(B)** Is a modified reproduction of three drawings by Schwalbe (1880) illustrating how the lateral side of the embryonic thalamus progressively deforms into a caudally oriented direction as a consequence of internal thalamic growth and parallel massive growth of the telencephalon and its stalk by the passage of numerous thalamocortical and corticothalamic fibers. Actually, the deforming (and enlarging) striped rostral thalamic boundary is not with the telencephalon, as imagined by Schwalbe, but with the prethalamus (compare **C**). Lack of understanding of this deformation stands at the basis of the erroneous idea of a *metathalamus* containing the medial and lateral geniculate bodies as derivatives of a neuroepithelial region immediately “posterior' to the main thalamus, which would have to be pretectal (see real topologic position of these primordia in Figure 7B, and comments in the text). **(C)** These two schemata were modified from originals in the work of Hochstetter (1919), who aimed to visualize the same thalamic deformation highlighted before by Schwalbe (1880). Hochstetter accepted like his predecessor the simplistic idea that the thalamus can directly contact the striatum (false assumption giving rise to the widely believed lamina affixa theory shown schematically in **A**). Modern prosomeric neuroembryology emphasizes the fact that the prethalamic part of the diencephalon is an intermediate neuromere rostral to the thalamus, which does not disappear (compare Figures 5, 10), and therefore *must* become intercalated at the thalamo-telencephalic transition in the fashion depicted in my changes to these schemata. The prethalamus (green and red areas) becomes deformed (both stretched radially and flattened rostrocaudally) into a rather thin territory placed all along the supposed interface of the thalamus with the telencephalon. The flattened prethalamus is depicted as a thin *green* band representing its dorsal pial surface that remains visible in dorsal perspective, accompanied by the similarly stretched dorsalmost prethalamic area, the red-labeled prethalamic eminence, also in dorsal perspective (PThE). The latter shows in this view both its original pial and ventricular surfaces, evaginated in part through the interventricular foramen into the medial hemispheric wall (what protrudes at the lateral ventricle next to the terminal sulcus, without needing any questionable adhesion, is the eminential prethalamus, and not the thalamus proper). The neighboring telencephalic basal ganglia are reinterpreted as including the lateral and medial ganglionic eminences (LGE in violet, MGE in light blue), and the MGE is further marked as containing periventricularly the BST complex, which contains in this view only the supracapsular stria terminalis. Finally, a line of black dots indicates the non-fimbrial sequential insertions of the forebrain chorioidal tela (see fimbrial insertion—fi- in **A**). The complementary chorioidal insertion begins at thalamic levels (in front of the epiphysis) with the *taenia thalamica* proper (tth; right side), which ends at the velum transversum fold, or thalamo-prethalamic border at the chorioidal roof (vel.transv.; represented by thick transverse black bar). We successively reach next the small *pre-foraminal* and *foraminal* parts of the *taenia prethalamica* (new term introduced here), which extend the chorioidal insertion into the interventricular foramen, now attaching at the eminential prethalamus. From there the dot line proceeds along a *post-foraminal* prethalamic taenial region (tpth; right side) that courses along the stretched red PThE up to its apparent “caudal” end (note this is no real rostrocaudal course, according to Schwalbe and Hochstetter, because it remains within the deformed prethalamus). This post-foraminal prethalamic taenia correlates in topography with the classic “thalamic” chorioidal sulcus (chs; **A**, right side; this was previously held to correspond to an apparent attachment at the border between the pars affixa and the pars libera of the fissural chorioidal tela—compare **A**-, but is reinterpreted here as being prethalamic and relating in depth to the prethalamic reticular nucleus—compare Figure 12; it is to be noted that, since chorioidal tissue is essentially roof plate, it is impossible that the thalamus has a second chorioidal roof plate apart of the one that covers the 3rd ventricle). Once the stretched PThE ends above the optic tract, next to the posterolateral pulvinar and the LG (right side), the continuation of the non-fimbrial insertion line of the chorioidal fissure apparently jumps here from the diencephalon (prethalamus) onto the telencephalon. It is only here where we can truly see a taenial attachment at the infracapsular BST (tail portion), or, finally, at the medial amygdala. Here it meets the amygdalar tip of the fimbrial taenia. This *sphenoidal taenia* decorates the sphenoidal horn of the lateral ventricle, participating with the fimbrial taenia in the final part of the chorioidal fissure. This attachment characteristically is no longer associated to the stretched eminential surface that covers the prethalamic reticular nucleus (compare Figure 12).

Importantly, the p3 or prethalamic prosomere completely separates the thalamic prosomere (p2) from the telencephalon and hypothalamus (Figures 1B, 5A, 10, 13A). This is an incontrovertible prosomeric conclusion that needs to be assimilated with its corollaries by any attentive modern neuroanatomic mind. Indeed, columnar literature frequently assumed that the thalamus directly contacts striatal telencephalic formations across the so-called “opto-striate, or thalamo-striate sulcus” (see Figure 11A taken from the 1979 edition of Gray's Anatomy); however, this classic “thalamus” really was the indistinct sum of alar thalamus and alar prethalamus (Figure 11C). The thalamo-striate sulcus, also known as sulcus terminalis, would roughly correspond to a *prethalamo-subpallial boundary*. While the prefix “thalamo” in the cited classic sulcus name is obviously wrong and means “prethalamo,” the suffix “striatal” is also wrong as regards the basal ganglion that establishes such “thalamic” contact, given that other subpallial parts of the telencephalon are now known to be nearer to the prethalamic diencephalon than the striatum (the latter is in fact most distant, being a derivative of the lateral ganglionic eminence; LGE; Figure 11C). The pallidal and diagonal subpallial areas are the elements derived from the medial ganglionic eminence that are closest to the diencephalon or, more precisely, to the prethalamus (Pal; Dg; Figure 10; MGE; Figure 11C; see our subpallium model in Puelles et al., 2013, 2016). They are represented at the ventricular surface by the lateral and medial bed nuclei of the stria terminalis, respectively; thus, the only really possible contact is between the *prethalamus* and the *diagonal area plus BSTM*, and certainly not the striatum. Interestingly, the classic authors clearly were not able to distinguish the derivatives of the embryonic medial and lateral ganglionic eminences even in advanced embryos, or perhaps were blocked in their thinking by the idea that all subpallium was striatal (e.g., Hochstetter, 1919, a major embryologist, in whose sections one often can see the darker and smaller pallidum domain); other classics failed at the same task for different reasons, e.g., because they wrongly assumed that the pallidal complex was hypothalamic (e.g., Christ, 1969; Kuhlenbeck, 1973).

As we now know, the transversal thalamic and prethalamic diencephalic wall regions, as well as the hypothalamus, were wrongly interpreted as longitudinal columns in the columnar model, which caused many confusing inconsistencies and conundrums (supposed “longitudinal” items found orthogonal to other longitudinal elements, or postulated “ventrodorsal” items found clearly parallel to longitudinal landmarks). In the prosomeric model, the *names* pretectum, thalamus and prethalamus are easily understood and consistently applicable with reference to all sorts of histologic material, if they are used strictly according to the respective alar domains of the p1–p3 prosomeres. Moreover, we also can apply the same easy terms to the whole segments when we loosely say “pretectal, thalamic or prethalamic segments, prosomeres or neuromeres.” In those expressions it is understood that we are adding the tegmental (basal/floor) portions of these units to the main alar components (Figures 10, 13A). We even find it is sometimes useful to employ allusively the expressions “pretectal, thalamic or prethalamic basal plate or tegmentum” (alternative to p1Tg, p2Tg, p3Tg).

## Specific Pretectal Issues

As regards the nomenclature of pretectal grisea there are no major semantic problems, because the axial references are here comparable in both models. Figure 8 shows a number of pretectal structures that classic literature tended to ascribe wrongly to the midbrain, notably the terminal nuclei of the basal or accessory optic tract, the classic posterior pretectal nucleus and the parvocellular red nucleus. There are otherwise problems due to our present very poor knowledge of the number of true pretectal nuclei in mammals, due to the region's classical Cinderella status, heightened by the undistinctive Nissl aspect of the mammalian pretectum (but see horizontal images in Figure 9; Puelles E. et al., 2012a, as well as recent work by Márquez-Legorreta et al., 2016). We are presently working on the mouse pretectum with genoarchitectonic markers, hoping to redress (partially, at least) this situation (Ferran et al., in preparation). The main semantic problem in the pretectum apparently was the incorrect “posterior pretectal nucleus” name, because this nucleus is instead distinctly mesencephalic, as commented above. The literature on mammalian visual projections mentions a nucleus of the optic tract, which is a term referring in my opinion to the retinorecipient superficial stratum of the classic posterior pretectal nucleus, though it is often used as if it was an independent pretectal entity. In order to erase the consequent confusion in the literature, we have proposed to name “*tectal gray*” the single rostral mesencephalic retinorecipient entity found rostral to the superior colliculus and caudal to the posterior commissure. This name and topographic ascription already existed in earlier comparative neuroanatomy of non-mammalian tetrapods (TG; Figure 9; review in García-Calero et al., 2002; Puelles et al., 2007, 2018).

## Specific Thalamic Issues

As regards the “thalamus” (alar p2), a term whose prosomeric meaning incorporates the habenular region (the columnar “epithalamus”) to the old “dorsal thalamus,” the modern view merely applies to its morphologic referent an oblique intrinsic dorsoventral dimension which is different from the columnar one (Figures 1A,B, 5, 7, 8, 10, 13A). This is so because at this point the natural forebrain length axis starts to bend together with the cephalic flexure (Figure 1B); usefully, the strictly dorsoventral course of the retroflex tract always marks the caudal thalamic border and the real dorsoventral direction at the back of the thalamus (rf; Figures 1B, 13A,B; Puelles et al., 2012b). This tract is compact and is seen only periventricularly. However, there exists as well a fiber-rich more lateral pretecto-thalamic limiting lamina that delineates the same boundary through most of the mantle layer. This fibrous lamina was first described, as far as I know, by Coggeshall (1964), in a curious non-neuromeric paper dealing with evident neuromeres in the rat, who called “posterior thalamic septum” the transversal fibrous laminar boundary of the thalamus. He clearly related it to the thalamo-pretectal interneuromeric constriction (his “middle thalamic fold”; [p2/p1 limit]). His material also reveals that the zli of Rendahl (1924) and Gilbert (1935) [the p3/p2 limit] represented his “anterior diencephalic fold,” while his “posterior diencephalic fold” was the pretecto-mesencephalic interneuromeric border caudal to the posterior commissure –[p1/m1 limit]; check pc in Figures 1B, 7B, 8, 9A, 13A: cp in Figure 4. Recently Márquez-Legorreta et al. (2016) have rediscovered this limiting septum in a chemoarchitectonic analysis of this area in the adult rat, calling it “pretecto-thalamic lamina,” after discussion of other references to it in the literature. Like the pretectum before, the more massive thalamus is also wedge-shaped, being longer dorsally than ventrally (Figures 1B, 10, 13A,B). This slight change in the spatial orientation of the dorsoventral thalamic dimension affects somewhat our appreciation of the relative topology of individual thalamic nuclei or nuclear complexes. For instance, columnar interpretation wrongly takes the medial geniculate body to be the “caudalmost” thalamic mass, when in fact it is the *ventralmost* thalamic mass, lying strictly ventral to the lateral geniculate body, as is readily seen in embryonic or any correctly interpreted adult material (LG; MG; Figures 7A,B); this is also confirmed by observing the topography of the well-known homologous entity in amphibians, reptiles or birds (Puelles, 2001; Puelles et al., 2007, 2018), a comparison unfortunately made difficult by the ancestral periventricular locus of the MG homolog in these lineages (Puelles, 2001).

The vague conceptual status of the pretectum as a caudal extension of dorsal thalamus, as well as the emphasis given by columnar authors to adult human relationships produced the now obsolete notion of the “metathalamus,” which would contain both the lateral and medial geniculate bodies in caudal proximity to (or identity with) the pretectum. Altman and Bayer (1995) unfortunately construed an aberrantly misguiding story about a pretended “methathalamic” (actually false pretectal) origin of both thalamic geniculate nuclei in the rat, which I had the opportunity to review critically in TINS by editorial invitation (Puelles, 1996). My relevant detailed comments did not obtain any contrary argumentative response from the authors. What happens with regard to the apparent “methathalamic” position of geniculate formations in primates is that the disproportionate growth of the thalamic mass in concert with the even more massive telencephalic growth and rotation deforms it unequally, so that its primary lateral surface (which carries the early-born and thus subpial geniculate bodies) is pushed backwards under the pulvinar, thus becoming oriented caudalwards, close to the independent pretectum (Figure 11B; this process was clearly illustrated by Gilbert, 1935; see also Figure 9 in Puelles et al., 2019, this book). This deformation due to differential growth was probably first pointed out by Schwalbe (1880), and was emphasized again by Hochstetter (1895, 1919), and a few other authors (however, none of these authors realized that the ventral thalamus or prethalamus also suffers a congruent deformation, with significant flattening of its mantle layer, due to its intercalation between telencephalon and thalamus; see Figure 11C). In the meantime, various other embryologists (e.g., Miura, 1933; Gilbert, 1935; Ströer, 1956; Coggeshall, 1964, and many others until present times), as well as comparative neuroanatomists, have concluded unanimously that the thalamic geniculate nuclei are both formed rostrally, next to the zona limitans intrathalamica. This explains why the thalamic lateral geniculate relates via the small intergeniculate leaflet to the prethalamic pregeniculate nucleus. The medial geniculate lies strictly *ventral* to the lateral geniculate primordium, as can be easily seen in nearly tangential sagittal sections through postnatal brains.

In a review (Puelles, 2001), I explored the possibility to explain the regionalized evolution of the whole thalamic mass into constant complexes or pronuclei out of which variable numbers of individual thalamic nuclei might evolve. The system stood on the basis of three (or perhaps four) dorsoventrally superposed “thalamic tiers” (dorsal, intermediate and ventral), understood as primordial pronuclei. It was held that these units retain evolutionarily some comparable connectivity (and other) properties in the thalamus of all advanced vertebrates. The cited three tiers are easily seen as individual cell masses in reptiles (Díaz et al., 1994; Dávila et al., 2000). Redies et al. (2000) and Martínez-de-la-Torre et al. (2002) examined them with molecular markers in the chick, where the intermediate tier acquires particular significance (review in Puelles et al., 2007, 2018). Indeed, individual tiers develop differentially in each lineage (eventually a tier involutes or grows disproportionately in some species). The dorsal tier (possibly complemented by a novel “associative” fourth tier) expands particularly in mammals correlative to evolutionary differential cortical growth (the potential fourth tier attending predominantly to associative cortex). The reference atlases and particularly the ontology I developed later for the public Allen Developing Mouse Brain Atlas (developingmouse.brain-map.org; offered since 2009) tried to show how the standard nomenclature for mammalian thalamic nuclei could be subsumed under the dorsoventral tier theory.

A further detail that recent molecular research has discovered relative to the thalamus, not contemplated by columnar schemata, is that the main thalamic mass consists largely of excitatory glutamatergic neurons. In rodents, inhibitory interneurons are visible only in the lateral geniculate nucleus, but other mammalian lineages including primates show them nearly everywhere, mixed with the thalamocortical projection neurons. It turns out that these cell types are produced separately. The thalamic alar domain first results patterned differentially into a thin anteroventral boomerang-shaped progenitor domain placed next to the zli core and the basal plate (av; Figure 13A) and a larger posterodorsal progenitor domain representing all the rest (pd; Figure 13A). The anteroventral domain is strongly influenced by the proximity of high SHH levels at the underlying basal plate and at the zona limitans (Figure 5B), resulting in a correlative *Nkx2.2* expression pattern at the av (and other forebrain areas under a similar influence, as shown in Figure 5C; by the way, this *Nkx2.2* band is the modern marker for the forebrain alar-basal boundary; discussion in Puelles E. et al., 2012a and Puelles and Rubenstein, 2015). The *Nkx2.2*-positive av domain has a differential molecular profile and fate compared to the larger posterodorsal *Gbx2*-positive rest of the thalamic progenitor layer (review in Puelles and Martinez, 2013, which also contains an hypothesis of how the zli organizer forms). Only the thin av domain produces inhibitory neurons, and it represents the source of the inhibitory neurons that secondarily invade tangentially the purely excitatory populations of the main posterodorsal thalamic mass, starting with the lateral geniculate nucleus. This tangential invasion is curiously selective with regard to the tiers, since the LG belongs to the dorsal tier, while the MG, which lacks such interneurons in rodents (Puelles et al., 2012b), belongs to the more precociously produced ventral tier (Puelles, 2001). A few inhibitory cells may invade the thalamic posterior periventricular nucleus through the thalamo-pretectal border. This distinction between differential progenitor domains of the thalamus according to functional cell type produced is not yet registered in any way in the standard nomenclature.

## Specific Prethalamic Issues

The prethalamus (alar p3) is another Cinderella-like area in the forebrain. Its intrinsic dorsoventral dimension is even more inclined than that of the thalamus relative to the brainstem axis, because of the cephalic flexure (PTh; Figures 1B, 5, 7, 8, 10, 13A). This diencephalic territory apparently was subliminally deemed less important than the thalamus because its neurons, which largely are of inhibitory nature (Puelles et al., 2012b), do not project into the telencephalon. The better known prethalamic derivative is the “thalamic reticular nucleus,” which already represents a semantic error; it manifestly lies within the PTh intermediate stratum (Figure 13A); for clarity, this well-known element should preferably be named “prethalamic reticular nucleus,” or simply “reticular nucleus.” Other prethalamic derivatives are the pregeniculate and subgeniculate retinorecipient nuclei (lying at the subpial stratum under the optic tract) and the zona incerta (across all strata, at the ventral end of the prethalamic alar domain; Figure 13A). The problem posed by the “zona incerta” is that many columnar accounts place it in the “subthalamus.” The latter concept is a misguided rest of its first introduction by Forel (1877), who referred it to the *basal* forebrain domain lying underneath the “thalamus.” His (1893, 1895, 1904) later renamed Forel's tegmental subthalamus as “hypothalamus.” As further historic steps led to expansion of the hypothalamus concept by aggregation of added alar plate subregions (review in Puelles L. et al., 2012a), some authors that apparently did not realize that the “subthalamus” term was already outdated tried to visualize a sort of fifth longitudinal column that could be called “subthalamus,” and which would lie intercalated between Herrick (1910) ventral thalamus and hypothalamus (this implies a shaky pentacolumnar version of the columnar model). Since such a fifth column strictly does not exist, or it would have been seen before, these attempts to construe a subthalamic column were condemned to compose the subthalamus out of parts taken either from the hypothalamus or from the ventral thalamus, or from both. We thus see literature placing arbitrarily in that virtual subthalamic region the alar prethalamic zona incerta and the alar “dorsal hypothalamic area” (“dorsal” here means in columnar parlance “close to ventral thalamus”). The only structure whose “subthalamic” identification is in some sense (*sensu* His, 1893) not contradictory is the subthalamic nucleus, because it is a migrated derivative of the *basal* hypothalamic retromamillary area (STh; Figure 10) and finally lies deep to the peduncle under the local alar plate, or thalamus *sensu lat*o of His (1893) (details in Puelles L. et al., 2012a; another name of this nucleus was for a time “hypothalamic nucleus,” showing that at the turn of the twentieth century “subthalamus” and “hypothalamus” were synonyms). Some embryological studies led authors to believe that distinct ventricular zone domains could be visualized for the subthalamus and the hypothalamus, but these turned out to correspond to the two hypothalamic prosomere domains identified by us (Puelles L. et al., 2012a; Puelles and Rubenstein, 2015); both are clearly hypothalamic. For clarity's sake, Puelles L. et al. (2012a) argued that we should eliminate altogether any continued use of the “subthalamus” terminology, excepting the individual *subthalamic and parasubthalamic nuclei*, which cause no problem and must be understood as basal hypothalamic formations (STh originates within basal and retromamillary PHy; Figure 10; later it migrates dorsalward, acquiring secondarily a position within the equally basal retrotuberal area; RTu; Figure 10). Conversely, the zona incerta must be firmly ascribed to the ventral rim of the prethalamus, or the ventral rim of alar p3 (zi; Figure 13A).

Finally, as mentioned above, the hyperdorsal subregion of the prethalamus forms the prethalamic eminence (PThE/pthe; Figures 10, 13A; see also Figure 6B for amphibians). A sizeable part of this region bends over through the interventricular foramen into the medial telencephalic wall, carrying with it its attached roof plate chorioidal tela and chorioidal taenia (a taenia is an insertion of a portion of roofplate chorioidal tela into the dorsal lip of the corresponding alar plate; see Figure 1B and thick black roof plate in Figure 10; also compare Figures 4, 5A of Lakke et al., 1988; I have decided to use what seems the etymologically correct orthography of this term, if it derives from the Greek *khorion or chorion*—vascularized fetal membrane, and thus leads to “chorioidal,” as used by numerous classic authors, but not so much by modern ones; Werner, 1956, p. 156). Accordingly, the prominent ventricular contour which we see delimiting the back side of the interventricular foramen (e.g., the calretinin-positive pthe; Figure 13A) is not the true dorsal end of this part of the prethalamus; the evaginated part lies hidden as a flap that extends beyond this eminential bulge within the immediate medial telencephalic wall, in the vicinity of the medial ganglionic eminence (mge; Figure 13A), and separated from the latter by the sulcus terminalis. The prethalamic chorioidal tela thus projects into the medial telencephalic wall, contributing to the formation of the supracapsular part of the classic chorioidal fissure. Given that most of the authors along these last 100 years have not been very much aware of the prethalamus (having misinterpreted the prethalamic eminence as a thalamic eminence), nor of its specific transition into the medial wall of the telencephalon via the prethalamic eminence, a general false belief was prevalent that the thalamus directly contacts and attaches to the subpallial (striatal) telencephalon (see Figures 11A,B). The neuroanatomists dealing with the chorioidal fissure generally failed to understand the local morphologic configuration (e.g., see Swanson, 2012 concept of the roofplate-derived fissure, by definition a longitudinal item, represented as a transverse structure). As a result of this confusion, a mythical, largely conjectural interpretation developed of what one sees at this obscure corner of the forebrain after dissection or sectioning, particularly in the human brain. This was the theory of the *lamina affixa*, an hypothetic, but really inexistent, piece of chorioidal tela believed to interconnect the telencephalic sulcus terminalis with the supposedly adjacent thalamus (referring in fact to what actually was misinterpreted prethalamus).

This theory states that the chorioidal tela that closes the telencephalic chorioidal fissure was originally wholly free of contact with the diencephalon and jumped from its clearcut fimbrial taenial insertion (border of the hippocampus; no problem with that) to another insertion at the stria terminalis, at the border of the *corpus striatum* (see left part of Figure 11A; note this is speculative, not real; nobody has shown a real section like this). Part of the fissural chorioidal tela would then adhere firmly to a neighboring part of thalamic pial surface, up to the so-called chorioidal sulcus (chs; right part of Figure 11A). The adhered part of chorioidal tissue would form the so-called *pars affixa of the fissure*, or *lamina affixa*, and, since this lamina is so thin, this supposedly causes a *pial* part of the dorsal thalamus to emerge under its covering at the floor of the lateral ventricle, just medially to the stria terminalis and the thalamo-striatal terminal sulcus (right side of Figure 11A); the non-adhered rest of the fissural chorioidal tela would be the *pars libera*, which would go on to form the chorioidal plexus of the lateral ventricle (Figure 11A). This theory is a conjecture, because the postulated adhesion process has not been demonstrated histologically in an embryonic series. However, this account is found in most neuroanatomy textbooks.

**Figure 12:**
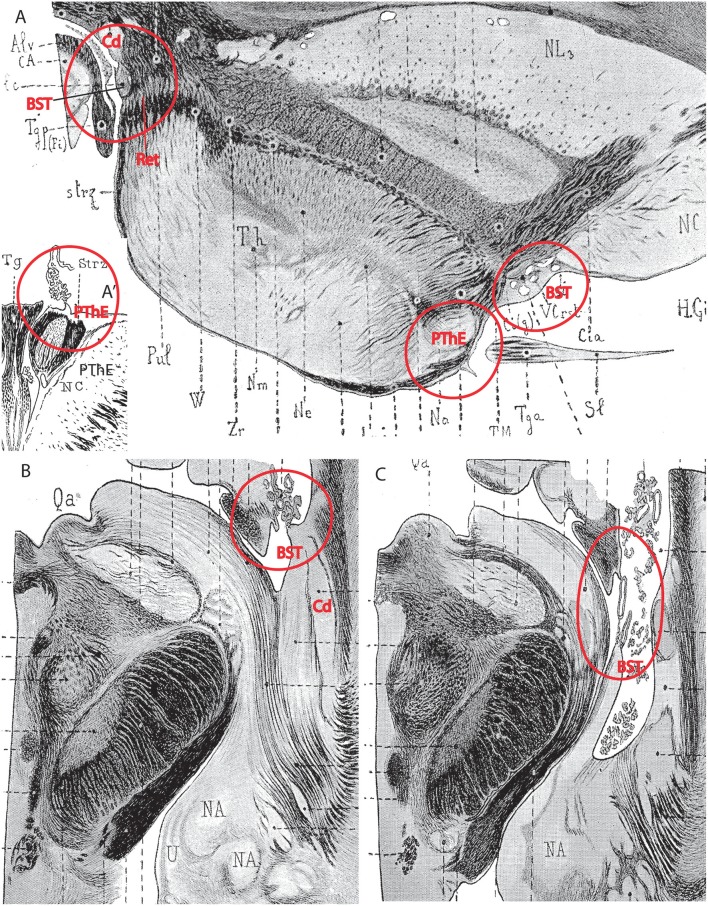
This plate brings together four separate drawings (or drawing details) modified by red annotations, taken from the work of the French neuroanatomist Dèjerine (1895) on the adult human brain (no copyright permission required). They illustrate my new interpretation of the roof-plate-derived chorioidal insertions (taeniae) associated to thalamic, prethalamic and telencephalic structures, as presented in Figure 11C (and negating what I call the *lamina affixa* myth). The Dèjerine (1895) work is one of the few places where this issue can be examined objectively in the adult human brain, because it contains very precise drawings of numerous sections in various planes. Other similarly useful material (not shown) is embryonic and can be found, e.g., in the human developing brain atlas of Hochstetter (1919), and other works showing abundant sections of appropriate embryonic material at high magnification. Dèjerine's drawings frequently depict the chorioidal tela insertions without deterioration, probably thanks to the celloidin embedding method used, which preserved the delicate chorioidal tela (normally torn or fragmentary in many published sources where sections from hand-dissected brains are shown). My thesis is that the classic lamina affixa theory (see text of Figure 11A) is a myth that emerged because classic authors confused parts of the prethalamus as being thalamic, and pallido-diagonal parts of the telencephalic subpallium as being part of the “corpus striatum,” and as a result misinterpreted the local massive morphogenetic distortions, including the appearance of part of the prethalamic eminence beyond the interventricular foramen. The prediction tested in these images is that the fissural chorioidal insertion classically held to be the transition of the same tela between *pars affixa* and *pars libera* (Figure 11B), and found beyond the interventricular foramen along the “thalamic” chorioidal sulcus, actually represents an insertion *along the prethalamic eminence* (which thus substitutes for the mythic thalamic ventricular bulge). This interpretation negates any adherence whatsoever; the surface classically interpreted as the *lamina affixa* covering the top of the thalamus would be a part of the ventricular surface of the prethalamic eminence that evaginates along the interventricular foramen into the lateral ventricle consequently to morphogenetic deformation of the prethalamus in the context of massive telencephalic evagination, growth and rotation. The deformed dorsal eminential prethalamus is systematically associated in depth with the reticular nucleus (also alar prethalamic, and crossed by the thalamo-cortical connections). Wherever the chorioidal taenia is prethalamic, one sees the reticular nucleus directly underneath, and this occurs along the whole chorioidal sulcus. However, once the prethalamus ends beyond the pulvinar, the fissural chorioidal tela apparently extends wholly into the temporal lobe of the hemisphere (as does the hippocampal fimbria), becoming fixed *now* at the subpallial locus of the *temporal* infracapsular bed nucleus of the stria terminalis, medially to the caudate tail (but not before, at the supracapsular BST, next to the caudate body and head, as was classically assumed). This new hemispheric insertion continues all the way to the medial amygdala retro-uncal region, where the fissure ends jointly with the fimbria. In **(A)** we see a detail of a horizontal section passing through the interventricular foramen, in which we can easily identify the pallidal and striatal basal ganglia, the internal capsule and the thalamus, which is surrounded throughout rostrolaterally by the reticular nucleus (prethalamus; marked Zr, zona reticularis, by Dèjerine, 1895). The red circle with the BST tag calls attention to the subventricular bulge of the BST complex next to the caudate head; it can be seen that there is no chorioidal insertion on it, though the stria terminalis tract and typical accompanying optostriate vessels are there (note also that the thalamus proper does not reach the BST, because the prethalamic reticular nucleus intercalates in between). The prosomeric model predicts that the thalamus never contacts any telencephalic component, being necessarily separated (even if tenuously) by some part of the prethalamus. The chorioidal insertion at the foraminal beginning of the chorioidal fissure appears instead at the slightly prominent neighboring locus circled in red and identified as the prethalamic eminence (PThE), some distance away from the BST locus. Classic authors uniformly interpreted the “thalamic eminence” as belonging to dorsal thalamus, namely as the anterior thalamic nucleus (and so did Dèjerine, 1895 as well), but developmental knowledge accrued in the meantime leads us to interprete this mass as prethalamic (the anterior nucleus lies slightly higher, as seen in other Figures of Dèjerine, 1895). Note a number of characteristic gene markers are present at this locus (see calretinin in Figure 13A and others in Shimogori et al., 2010). Since the chorioidal fissure is a telencephalic formation, albeit continuous with the diencephalic chorioidal roof, and at some levels the insertion occurs at the BST locus (as we will see below), classic authors speculated that the insertion at this rostral foraminal level also was at the BST bulge irrespective that one could not see it. This led to using the general explanation believed at the time that a section of the predicted chorioidal tela adhered to the neighboring “thalamic surface,” forming the so-called *lamina affixa*. However, as we see, proper analysis shows there is no “thalamus,” but only *prethalamus*, between the rostral BST and the foraminal chorioidal insertion at the PThE. What was supposed to be thalamus covered by *lamina affixa* is in fact standard ventricular surface of evaginated PThE that protrudes in a diminishing gradient beyond the interventricular foramen, all the way to the area next to the pulvinar (note that the pulvinar belongs jointly with the geniculate nuclei to the true lateral wall of the thalamus; Figures 11B,C). This rostral PThE protrusion is seen also in a coronal section passing through the same locus in the inset **(A′)** (red circle at left with PThE tag). The fiber packets seen within the PThE in **(A,A′)** probably represent components of the stria medullaris, which courses through the PThE into the more caudal thalamic habenula. In coronal sections placed all along the thalamus between its rostral and caudal poles we would see that the insertion line along the chorioidal sulcus is always precisely associated in depth to the point where the reticular nucleus approaches the brain surface (not shown; see any such section in Dèjerine, 1895). However, in the section shown in **(A)**, we also see behind the caudal pole of the pulvinar another chorioidal insertion (also emphasized by a red circle). This non-fimbrial taenial insertion jumps at the center of the caudal red circle from the hippocampal fimbria marked Tgp (Fi) into a small telencephalic area originally labeled “lc,” that is, “lamina cornea,” an old name for the supracapsular BST (BST in red). This new insertion lies just outside the reticular nucleus band surrounding the thalamus [Zr]. The classic lamina cornea is what we now identify as the BST, a derivative of the medial ganglionic eminence which accompanies the striatal tail of the lateral ganglionic eminence (Cd in red; the tail appears as a white mass above the lamina cornea or BST). Here we see a non-fimbrial taenial insertion that no longer occurs directly on top of the reticular nucleus, but lies immediately outside it, associated truly to the telencephalic BST formation, medially to the caudate tail (white round mass seen above it). This image thus practically shows the locus where the prethalamic PThE chorioidal insertion ends and a sphenoidal telencephalic BST insertion begins. **(B)** Shows a somewhat lower horizontal section through the ventral pulvinar (still above the geniculate bodies) and **(C)** passes through the ventral part of the internal capsule and the medial amygdala/uncus area (NA, U in **B**); the red circle indicates that the fissural chorioidal insertion continues attached to the sphenoidal BST portion, next to the caudate tail (Cd). The section in **(C)** is slightly more ventral and the red circled area shows the chorioidal insertion finally reaching the medial amygdala (labeled NA).

I proposed years ago in a conference on human brain development held in Rome that consideration of the obligatory presence of prethalamic derivatives in that scene showed the lamina affixa theory to be an unnecessary myth, since the observed morphologies and relationships could be explained alternatively, without recurring to undemonstrated adherence between telencephalic and thalamic pial surfaces. Figures 11, 12 (and their legends) collect my position and some evidence supporting it.

I basically suggest that we can distinguish three successive parts of the *prethalamic chorioidal tela*, which derives from the p3 roof plate, and attaches primarily to the hyperdorsal alar PThE (Figures 1B, 10): (1) a small *pre-foraminal* part is found just caudal to the interventricular foramen; it includes the chorioidal tela closing the rostral diencephalic part of the third ventricle, and it jumps from the pre-foraminal prethalamic taenia into its contralateral homonym; this rather small part probably forms the rostral arm of the *velum transversum*, since the zli, the interthalamic p3/p2 boundary, ends dorsally at the velum (a transversal fold in the chorioid roof plate, classically interpreted as tel-diencephalic limit, but corresponding in fact to p3/p2 zli boundary; h.s-t.r.; v.t.; in Figure 4; VEL.TR. in Figure 6; see also Lakke et al., 1988; their Figure 5A; vel.transv. in Figure 11C, right side). This small pre-foraminal prethalamic taenial sector is usually misidentified as part of the “thalamic taenia” (term that should be restricted to p2, that is, to areas caudal to the velum transversum; see tth in Figure 11C); (2) a small *foraminal* portion of the prethalamic taenia is next found above the interventricular foramen itself and the major intraventricular bulging portion of the PThE (Figures 11C, 12A,A′); here the taenia relates to the chorioidal roof of the interventricular foramen; the local chorioidal tela probably jumps from the foraminal prethalamic taenia into the taenial insertion at the back of the subfornical organ and the hippocampal commissure; (3) finally, there is a longer *post-foraminal* art of the prethalamic taenia, whose insertion runs along the thalamic “chorioidal sulcus,” which really represents the free dorsal lip of the deformed prethalamic eminence (i.e., the “thalamic chorioidal sulcus” is really a stretched *prethalamic* insertion site). The real nature of the morphogenetically stretched PThE is revealed because it correlates systematically with the linear band where the deeper prethalamic reticular nucleus maximally approaches the brain surface (dot line over the red PThE in Figure 11C; see legend and images in Figure 12; this relationship was never recognized before). This longer post-foraminal portion of the prethalamic chorioidal tela jumps across the fissure from its stretched PThE insertion to the opposed fimbrial supracapsular taenia (and, accordingly, is *not* inserted in the area of the stria terminalis, but in the PThE). The surface classically interpreted as *lamina affix*a covering the “thalamus” extends between the post-foraminal prethalamic taenia and the stria terminalis, next to sulcus terminalis. This surface is ventricular and represents the evaginated trans-foraminal ventricular surface of the PThE participating in the medial wall of the hemisphere, or the floor of its lateral ventricle, up to the sulcus terminalis.

After the chorioidal sulcus and the prethalamic chorioidal tela both finish close to the caudal thalamic pole, apparently at the lateralmost part of the caudal pulvinar, not far from the underlying lateral geniculate nucleus, and, more precisely, next to the prethalamic pregeniculate nucleus (Figure 11C), there continues a purely telencephalic part of the non-fimbrial fissural chorioidal taenia, the final, or *sphenoidal taenia* sector. Here we see the chorioidal fissure tela jumping from the fimbria to an extra-diencephalic taenial attachment at the *sphenoidal (infracapsular) BST* and later at the posterodorsal medial amygdala (isolated dot line marked tst in Figure 11C; compare BST; Figures 12A–C). In humans the fimbrial taenia ends at the uncus, next to the dentate gyrus; see dissection data obtained by Klingler (1948).

Villiger and Ludwig (1946) and Villiger et al. (1951) are the only authors who considered this prethalamic taenial issue, in context with the known torsional morphogenesis of the hemisphere around its stalk, which brings the primitively posterior temporal pole into a more anterior position, particularly in large-brained mammals. They thought that the prethalamic chorioidal roof plate might be stretched as far as the uncus and medial amygdala, but I doubt this interpretation because an even more stretched PThE and reticular nucleus would be expected then to reach as well the amygdala, which does not happen, apparently.

## Problems With the Hypothalamus

The hypothalamus is the forebrain site where the columnar-inspired conventional terminology of the last 100 years is most conflictive with the prosomeric concepts, due to the blatant difference in the respective axial references (90° of difference; i.e., the columnar *length* axis corresponds to the prosomeric *dorsoventral* dimension). So far a complete alternative nomenclature with a consistent prosomeric terminology has not been proposed. I worked on it while writing the Puelles L. et al. (2012a) chapter, but finally abandoned this effort, thinking it would require too many changes, and, therefore, also demand too much from the receiving end. It seemed best for clarity to momentarily keep most conventional names (with sparse novelties or adjustments), while we emphasized the topologic and causal interpretation advantages derived from the prosomeric model and its molecular underpinnings, such as a dorsoventral molecular patterning partially shared with more caudal forebrain regions (comments on this in Puelles and Rubenstein, 2015). Along with this idea we postulated two prosomeric units within the redefined hypothalamus (hypothalamic prosomeres 1 and 2, or hp1, hp2; numbered in caudorostral order, like in the diencephalon; see our rationale for this in Puelles and Rubenstein, 2015). The idea was to first try to win over the readership with our theoretic morphologic analysis, and later let the field address gradually, with only occasional help from our side, the problem posed by the incongruent columnar anatomic descriptive terms. The major scientific advantage of the prosomeric model of the hypothalamus is that it allows causal analysis within a framework of patterning mechanisms that is common for the whole forebrain as far back as the isthmo-mesencephalic boundary. This desirable aim absolutely needs correcting the arbitrary and aberrant decision taken by Herrick (1910) on the axial reference. Once this is done, standard anatomic descriptors will have changed meanings and will need to be adapted to the correct axis. The field will find the how, when, and who to do it.

I already covered above the general position of the hypothalamus relative to the diencephalon (and the prethalamus in particular). A second basic point to attend is the hypothalamo-telencephalic border. Columnar convention during the second half of the twentieth century has held that the hypothalamus includes “rostrally” the preoptic region. This was initially not so, particularly when the “hypothalamus” term was first defined by Forel (1877) and His (1893, 1895, 1904), referred exclusively to a basal plate entity (Figure 2A). However, other authors later incorporated alar regions to the hypothalamus concept, as it stands at present (historic review in Puelles L. et al., 2012a), and that tendency eventually also led to a tentative joint description of the preoptic area with the hypothalamus (e.g., Le Gros Clark, 1938, p. 9: “Although, strictly speaking, this area is no part of the hypothalamus……, it requires to be described briefly here because the two cannot be separated morphologically”). Later the step was taken to adopt its straightforward ascription to the hypothalamus (McRioch et al., 1940; Christ, 1969). One unifying morphologic principle apparently was the shared third ventricle relationship. Notably, the magnocellular cells of the paraventricular and supraoptic nuclei were first classified as preoptic (i.e., telencephalic), and only later thought to be hypothalamic.

Once the molecular era began, it turned out that the preoptic area shows distinct similarity in gene expression patterns (and causal mechanisms) with the adjoining telencephalic subpallium (moreover, many preoptic neurons migrate tangentially into the telencephalic subpallium and pallium, a feature characteristic of subpallial domains, and none of its derivatives move into the hypothalamus). In parallel, there is a sharp molecular boundary between the preoptic area and the neighboring hypothalamic paraventricular area (Flames et al., 2007; Shimogori et al., 2010; Puelles L. et al., 2012a; Puelles and Rubenstein, 2015). Again in this case, the judgment of pre-columnar experts turned out to be correct in the long run, and the relevant conclusion appears incorporated in the prosomeric model, namely the ascription of the preoptic area to the telencephalic subpallium. Obviously, this boundary is interpreted as a dorsoventral (longitudinal) one within the prosomeric model, insofar as the whole telencephalic field develops within the dorsal part of the hypothalamic alar plate (Puelles and Rubenstein, 2015).

The transition into the telencephalon, however, is double, because we have two hypothalamo-telencephalic prosomeres (hp1 and hp2; *loc.cit*.). The sum of alar and basal hypothalamic domains of these prosomeres were newly named *peduncular hypothalamus* (PHy) and *terminal hypothalamus* (THy), respectively (Figure 10; Puelles L. et al., 2012a). I thought that these terms were needed immediately, to provide a clear prosomeric way to move around in the hypothalamus, pending full terminological corrections. The new terms evaded continuous discussion about the meaning of the descriptors “dorsal, ventral, rostral, caudal.” These terms are descriptive and easy to remember. “Peduncular hypothalamus” refers to the selective (dorsoventral) course of the *cerebral peduncle* through the PHy (see Figure 7B; also various relevant images in Puelles L. et al., 2012a and Puelles and Rubenstein, 2015). The peduncle only bends caudalwards when it reaches the basal plate of PHy, passing around the subthalamic nucleus (e.g., Figure 6B). This peri-subthalamic peduncular bend is readily visible in rodents and other small mammals, but not in the human brain, where massive peduncular growth results in an *apparent* straight course of the hypothalamic peduncle into the pes pedunculi.

On the other hand, “terminal hypothalamus” refers to the topologic *terminal position* of this region at the rostral end of the neural tube. Another related neologism that I introduced in Puelles L. et al. (2012a) was the “*acroterminal area*,” a name needed for the distinct bow-like vertical border of THy at the rostralmost end of the hypothalamus (it extends from the rostromedian mamillary body to the median septopreoptic crossing point of the anterior commissure (Figure 10; this latter locus was settled as being preoptic because its ventricular cells selectively express *Shh*, a feature not found outside the preoptic area; Puelles et al., 2016). The acroterminal area is an unpaired *transversal* entity, with floor, basal, alar and roof parts, oddly as it seems, and shows throughout its height (we must fight the psychological tendency to think of this height as a length) unique morphological characteristics, i.e., formations not existing elsewhere in the hypothalamus and the whole brain. These bespeak of a series of singular basal and alar prechordal inductive effects, which give rise to the neurohypophysis and median eminence, the anterobasal and chiasmatic areas, the vascular organ of the lamina terminalis, and the lamina terminalis itself, ending at the anterior commissure median crossing bed itself (see further details on this area in Puelles L. et al., 2012a; Ferran et al., 2015; Puelles and Rubenstein, 2015). There existed no earlier columnar term for this singular neural wall locus.

The terminal hypothalamus thus transits dorsalward into the unevaginated preoptic telencephalon, while the peduncular hypothalamus transits similarly into the evaginated hemisphere. The caudal limit of the subpallial preoptic region with the neighboring diagonal area relates to the end of the strong preoptic ventricular zone expression of *Shh* (Puelles et al., 2016; Puelles, 2017), as well as with the course of the fornix tract (Puelles and Rubenstein, 2015). This implies that the PHy must contact at this border with a different subpallium component, namely the diagonal area, which jointly with the pallidum forms the medial ganglionic eminence (Puelles et al., 2013, 2016).

**Figure 13:**
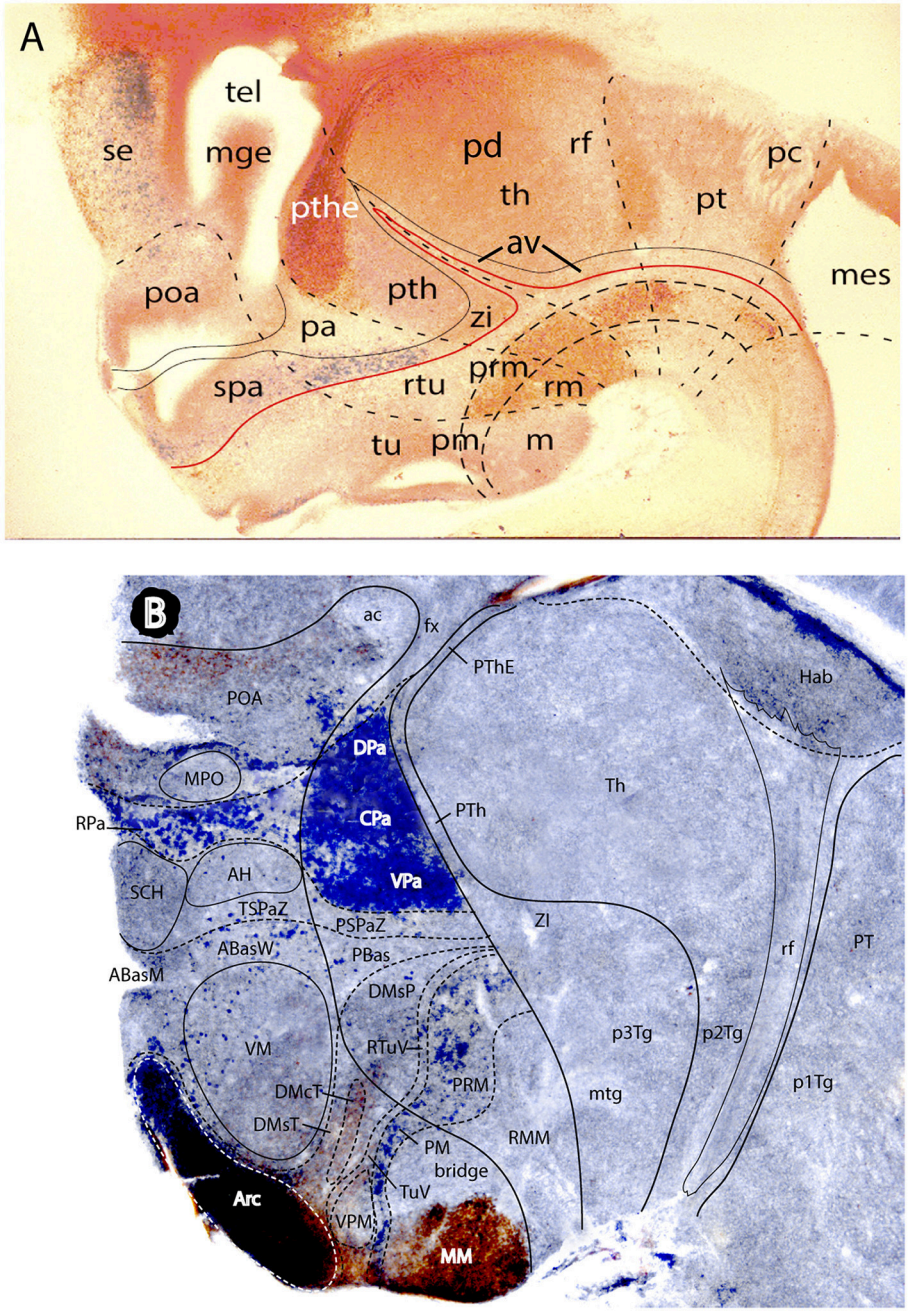
Two mouse brain sagittal sections reacted genoarchitectonically to visualize prethalamic and hypothalamic subdomains in a broader forebrain context (particularly diencephalic). Material extracted and slightly modified from (Puelles L. et al., 2012a) no copyright permission required). **(A)** Is a E16.5 mouse embryo sagittal section showing weak Dlx5/6-LacZ blue reaction at sites of *Dlx* gene expression e.g., the hypothalamic subparaventricular area (spa), continuous caudally with the prethalamic zona incerta area (zi), the latter being also continuous over the *Nkx2.2*-positive shell domains of the zona limitans (compare Figure 5C) with further alar areas always next to the basal plate down to the midbrain (this band coincides with sites producing GABAergic neurons). Calretinin immunoreaction was combined in **(A)** (brown reaction), to highlight the prethalamic eminence subregion (pthe) as well as some partial basal plate patterns across hp1, dp3, and dp2. The dash lines mark interprosomeric boundaries running from roof to floor. Note retroflex tract (rf) and posterior commissure (pc) as boundary-related landmarks. The alar prethalamus appears accordingly divided into three dorsoventral subdomains, namely the prethalamic eminence (pthe), the subjacent area occupied by the major prethalamic derivatives (reticular nucleus, and the pregeniculate and subgeniculate visual centers—marked “pth”) and the zona incerta area (zi). Note the underlying prethalamic tegmentum (separated by the red alar-basal boundary) is also divided into three dorsoventral subdomains (longitudinal dash lines), which also extend throughout the whole forebrain. The hypothalamic alar plate, in contrast, is divided only into two dorsoventral zones, the paraventricular area (pa) and the subparaventricular area (spa). The pa has a sharp molecular boundary with the alar prethalamus (pth); see also **(B)**. It is larger dorsoventrally within hp1, whereas the spa is larger within hp2 (peduncular vs. terminal hypothalamus; compare Figure 10). The basal plate hypothalamic territory is divided dorsoventrally in three longitudinal zones: tu/rtu (tuberal and retrotuberal; this expands rostralwards into the acroterminal area; check Figure 10), pm/prm (perimamillary/periretromamillary), and m/rm (mamillary and retromamillary). The preoptic area (poa) falls inside the telencephalic subpallium, jointly with the ganglionic eminences (here only the mge is seen) and the subpallial septum (se). **(B)** This sagittal section of an adult mouse brain shows hypothalamic molecular domains complementary to those labeled in **(A)**. The blue signal corresponds to Otp-LacZ reaction present in neurons that express the *Otp* gene. This signal is mainly characteristic of the oxytocin/vasopressin neurons of the hypophysiotropic magnocellular paraventricular nucleus. Here we see even more clearly than in the embryo **(A)** that there are quantitative differences between the paraventricular derivatives in PHy (the principal paraventricular nucleus; subdivided by Puelles L. et al. (2012a) into dorsal, central and ventral parts- DPa, CPa, VPa- which show differences with other markers) and THy (dorsal to unstained SCH and AH subparaventricular nuclei); the Pa area of THy contains less important paraventricular populations, but displays preponderant presence of the subpial supraoptic nucleus cells. Another site where islets of Otp-LacZ reaction are found is the basal pm/prm band, here again of larger size in PHy than in THy. Other Otp-expressing cells lie around the ventromedial nucleus (the VM shell), or are mixed with other neurons at the arcuate nucleus (Arc). The latter formation appears strongly counterstained with anti-NKX2.1 immunoreaction (brown), which extends somewhat into the neighboring terminal dorsomedial nucleus subregion (DM-T) and into the medial mamillary body (MM). The diencephalon shows a well-developed habenular complex (Hab), with the descending retroflex tract in characteristic prosomeric position (rf). Note also considerable adult compression of the periventricular stratum of the alar prethalamus (PTh, PThE), always intercalated between thalamus and hypothalamo-telencephalic structures.

**Figure 14:**
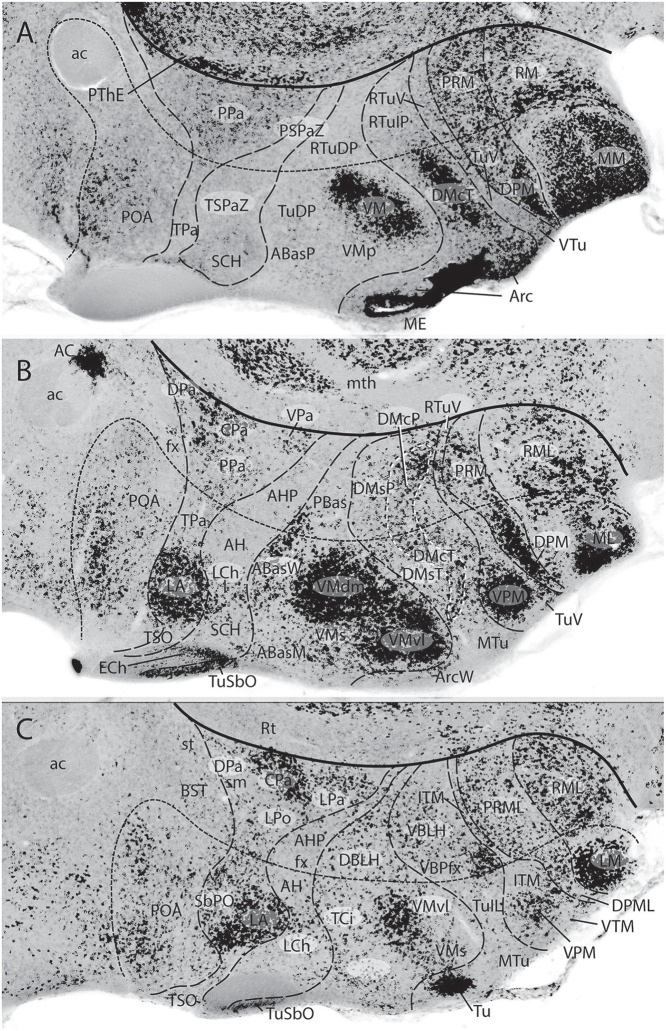
Three sagittal sections through the adult mouse hypothalamus, shown in medio-lateral sequence **(A–C)**, which were *in situ* reacted for *Glu2*, a gene marker of glutamatergic excitatory neurons (from Puelles L. et al., 2012a; no copyright permission required). The thick black line indicates the hypothalamo-diencephalic boundary (dorsal to the left; caudal to the top). The nearly parallel line with minute dashes is the *intrahypothalamic* segmental boundary that separates PHy from THy (or hp1 from hp2). Orthogonal dash lines separate alar and basal longitudinal progenitor domains. The images immediately make clear that the abundance of glutamatergic neurons is substantially higher at THy than at PHy **A–C**; this is part of the evidence why these domains are considered different prosomeres; some columnar authors described similar compartments as “dorsal and ventral” hypothalamus regions, whereas some other columnar authors identified them as dorsoventrally related subthalamus and hypothalamus columns (the PHy contains the subthalamic nucleus in sections lateral to **C**). Within the paraventricular alar area, numerous excitatory neurons appear aggregated at the lateral anterior nucleus (LA) within THy **(B,C)**; there is also a dense patch selectively at the CPa subnucleus in PHy **(C)**. The subparaventricular area is largely devoid of this cell type (SCH, AH, AHP). The massive bipartite glutamatergic population within the VM hypothalamic complex **(A–C)** contrasts with the overall scarcity found at the neighboring DM formation, excepting the latter's periventricular positive core population seen in **(A)** (probably migrated; DMcT, DMcP). The ventral and dorsal premamillary populations (VPM, DPM) also are largely excitatory **(A,B)**, as are the mamillary and retromamillary formations (MM, LM, RM, RML; **A–C**). Note the subpallium found dorsal to the hypothalamus (e.g., POA) is generally poor in glutamatergic neurons, as is the reticular prethalamus (Rt; **B,C**). In contrast, the prethalamic eminence only contains glutamatergic neurons (PThE; **A**).

**Figure 15:**
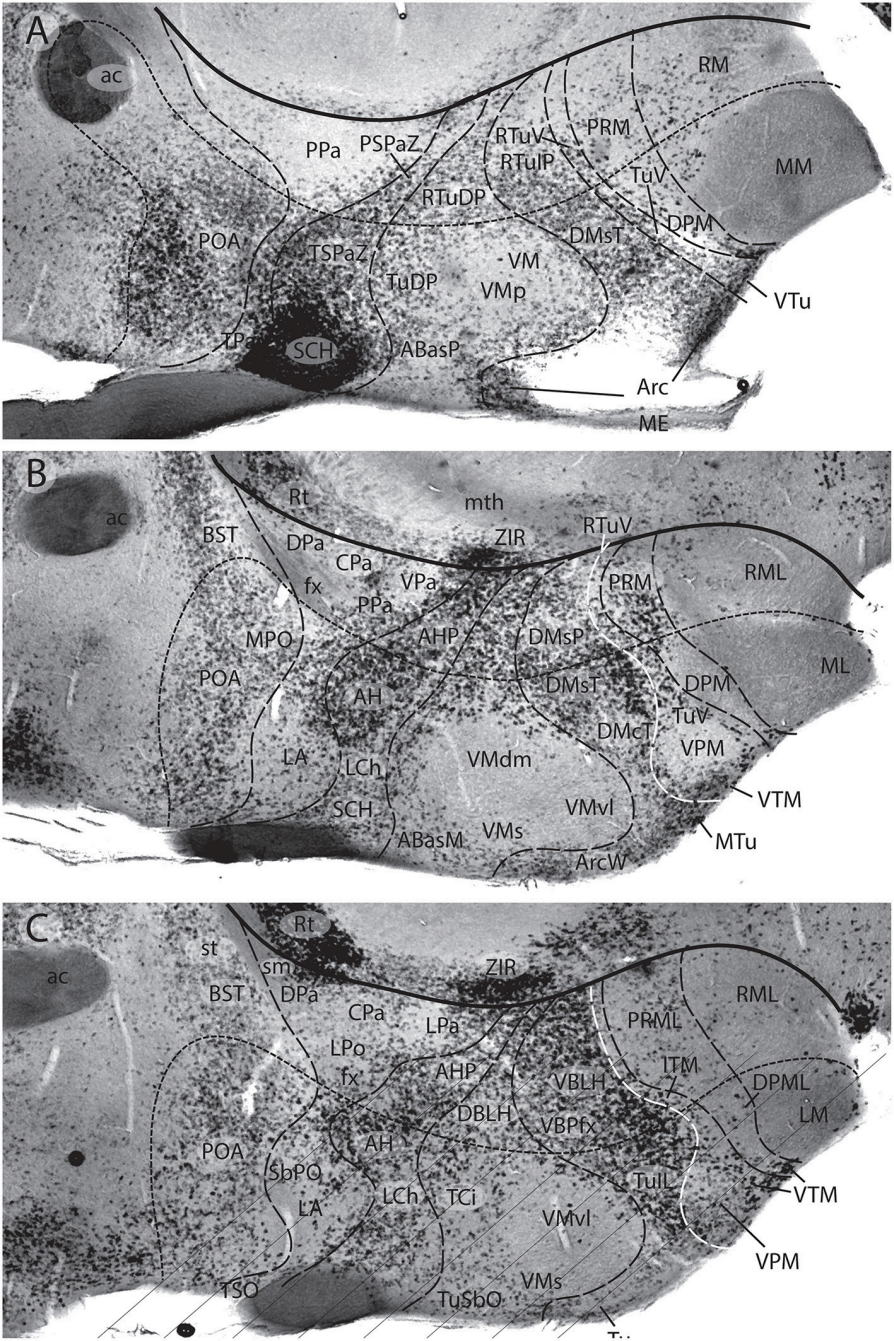
Three sagittal sections adjacent to those in Figure 14, through the adult mouse hypothalamus, shown in medio-lateral sequence **(A–C)**. They were *in situ* reacted for *Gad67*, a gene marker of GABAergic inhibitory neurons (from Puelles L. et al., 2012a; no copyright permission required). The thick black line indicates the hypothalamo-diencephalic boundary (same overall orientation as Figure 14). The nearly parallel line with minute dashes is the intrahypothalamic segmental boundary separating PHy from THy (or hp1 from hp2). Orthogonal dash lines separate alar and basal longitudinal progenitor domains. The overall image readily shows that the hypothalamic formations with marked numbers of excitatory neurons (Figure 14) have few if any GABAergic neurons (e.g., check LA, also most of the principal Pa nucleus, VM, VPM, DPM, MM, ML, LM; RM, RML—**A–C**). The maximal presence of GABAergic neurons appears at the SCH nucleus, and less markedly at the neighboring AH and AHP, all of them subparaventricular derivatives. The full dorsomedial formation across both THy and PHy is also rich in GABAergic neurons. The prethalamus (e.g., Rt; ZIR) also emerges as a GABAergic territory **(B,C)**. There is also a shell of GABA cells around the VM nucleus **(A–C)**. The preoptic area (POA) is also well provided with GABAergic neurons **(A–C)**. Seeing the curved and topographically oblique (deformed) boundary lines that separate paraventricular from subparaventricular alar entities, as well as basal formations, one understands that the habitual atlas coronal sections are not helpful in understanding these alternative distributions highlighted by the selective molecular markers and more appropriate sagittal section planes.

The major constituents of the alar regions of both THy and PHy are represented by two genoarchitectonically and chemoanatomically quite distinct longitudinal domains (with various shared markers across THy and PHy i.e., across hp1 and hp2; Figures 10, 13A,B; but see differential markers in Ferran et al., 2015). The relatively dorsal subdomain is the already mentioned paraventricular area, where magnocellular and parvocellular cell populations of the paraventricular nucleus and the supraoptic nucleus are produced (Figure 13B). These are glutamatergic and peptidergic neurons (probably excitatory neurons throughout; Figures 14A–C), and the area can be subdivided dorsoventrally into three subzones showing some differential characteristics (DPa, CPa, VPa; Figure 13B; Puelles L. et al., 2012a). The detailed topographic terminology evolved within the columnar interpretation for such supraopto-paraventricular cell populations is quite complex, with the added problem that individual cell groups were conceived along the logic of “potatoes in a potato sack,” i.e., without any histogenetic or patterning ordering principle. This is a general problem with columnar hypothalamic schemata, where constituent nuclei are illustrated literally as an elongated heap of variously sized balls devoid of developmental positional rules (e.g., Krieg, 1932; Ströer, 1956; Swanson, 2012). Indeed, columnar theory of the diencephalon did not even use alar and basal subdivisions, and considered the four postulated columns (Figure 1A) as homogeneous units at least for functional purposes, if not histologically (Herrick, 1948; Kuhlenbeck, 1973). Columnar authors in general, perhaps because of their emphasis on functions, did not postulate any developmental unit analogous to progenitor areas or histogenetic migration areas extending physically from the ventricle to the pial surface as a step in eventual differential columnar maturation. Indeed, the emergence of a multitude of nuclei in the thalamus or the hypothalamus occurs as if by magic (compare recently Alvarez-Bolado and Swanson, 1995; text p.24); mechanistic partitioning concepts leading to present-day progenitor zones evolved only within neuromeric models (e.g., Palmgren, 1921; Rendahl, 1924; Bergquist and Källén, 1954; Vaage, 1969, 1973; Puelles and Martínez de la Torre, 1987; review in Nieuwenhuys and Puelles, 2016), but were not used until the molecular era corroborated them, because of the columnar dogma that neuromeres supposedly did not exist. The lack of any positional logic for the multiple entities differentiated in 4 strata (see Puelles L. et al., 2012a, their Figures 8.30–8.33) makes any columnar hypothalamus map quite chaotic when examined in detail, a problem which is made worse by the novel evidence of numerous neuronal tangential migrations happening in various directions (review in Díaz et al., 2015; I refer to cells produced strictly at some hypothalamic subdivision which move developmentally into several adult positions; that poses a different sort of terminological problem). In practice, individual columnar supraopto-paraventricular cell groups were described as lying rostral to some preoptic formations, where others were placed caudal to them, but were also partly thought to lie caudal to some tuberal (basal) formations (e.g., as described by Swanson, 1987). Such chaotic positioning allowed by the columnar model (compare the correlative prosomeric mapping in Figure 13B) resulted apparently from the vagaries of the section plane employed and the intrinsic morphogenetic deformations of the histogenetic units observed using the prosomeric model (Figures 5, 10, 13–15). We accordingly proposed our much simpler schema of the paraventricular area with differential size across THy and PHy (Figure 13B), with a minimum of individual name changes, to make sense in our own descriptions (see Puelles L. et al., 2012a). We also distinguished different strata of the same area and derivatives that migrate tangentially away.

The second longitudinal domain of the alar hypothalamus lies immediately dorsal to the alar-basal boundary and underneath the paraventricular area (both across THy and PHy). This territory produces (in contrast to the suprajacent paraventricular area) mainly inhibitory GABA-positive neurons, a point inexplicably not commented by recent columnar authors. There is no theory nor discussion whatsoever about how a column (as defined within the columnar model) might produce separate groups of excitatory and inhibitory neurons (the fact is that if it is a really homogeneous unit, the column should not produce these distinct types of neurons). However, such alternative cell type distributions occur locally at various parts of the hypothalamus (see Figures 14, 15), consistently with differential genetic profiles enabling specific areas defined in the prosomeric model to produce either excitatory or inhibitory neurons, as happens elsewhere in the brain (subsequent tangential migration may intermix partially these separately produced populations). This clearly means that the columnar hypothalamus is not a real histogenetic unit (Puelles L. et al., 2012a; Díaz et al., 2015).

The name that immediately occurred to us for this mainly inhibitory alar hypothalamic band was the “*subparaventricular area*.” Luckily, this term had been already introduced precisely at this hypothalamic level, though in a more restricted sense, by the columnar authors Watts et al. (1987). We were happy to absorb it as an exercise in conciliation, and expanded it into its use in our model (SPa; Figures 10, 13–15; Puelles L. et al., 2012a). This area contains rostrally the acroterminal optic chiasma and is continuous caudally with the prethalamic zona incerta (spa; Figure 13A). This domain had no previous columnar name, due to the reasons commented above, but some well-known potato-like nuclei belong to it (e.g., the suprachiasmatic nucleus, SCH, and the anterior hypothalamic nucleus, AH/AHP, jointly with some less important elements). The “supra-”prefix in “suprachiasmatic” is inexact, because the nucleus is merely deep to the chiasma (i.e., nearer to the ventricle), not dorsal to it, but the SCH nucleus is so distinct in any case that this semantic difference seems not excessively problematic (this comment applies also to the “supraoptic commissures”). Similarly, the “anterior” descriptor in “anterior hypothalamic nucleus” happens to be acceptable in prosomeric interpretation, because the AH nucleus lies within the subparaventricular THy, which topologically is an *anterior* hypothalamic position also in our model. This term was actually used ambiguously by columnar authors, since they applied it to a nucleus that lies at the middle of the hypothalamus (particularly, if you believe that the preoptic area is an anterior hypothalamus part, as many authors still assume). This occurs because there was an historic time when the columnar hypothalamus ended at the AH, while its supraopto-paraventricular neighbors were still preoptic (i.e., telencephalic). This nucleus thus was for a time the most “anterior” part of the hypothalamus according to the columnar axis. Later, when the columnar model added the preoptic area to the hypothalamus, various authors (some in major atlases) started to identify the “anterior nucleus” partly or wholly inside the preoptic area (reviewed in detail in Puelles L. et al., 2012a), but nobody apparently noticed this error in topographic consistency. This example shows that a morphologically inaccurate descriptor (or an innocent descriptor used within an inaccurate model) tends to have perverse practical consequences, because sooner or later there are users that naively confide in the *apparent meaning* and are led astray into wrong conclusions.

Trying to be conservative, we largely used for the basal hypothalamic region names that already were in use before. We found basically three longitudinal zones stacked dorsoventrally across basal THy and PHy (Figures 10, 13). We distinguished dorsally a revised *tuberal/retrotuberal region* (Tu/RTu) and ventrally a *mamillary/retromamillary region* (M/RM), separated by an intermediate basal band, again distinct molecularly and structurally from Tu/RTu and M/RM, but previously unnamed, or very badly named, which we termed *perimamillary/periretromamillary area* (PM/PRM) (see Figures 13A,B).

As regards the change to “retromamillary”, it seemed ridiculous to continue describing as “supramamillary” a zone lying adjacent to the floor plate, caudally to the mamillary body (RM; Figure 10). Columnar authors always passed lightly over the fact of a *floor* plate present at the pretended *caudal* end of a column, since it could not be explained; it was inconsistent with the model. They would have preferred a direct axial continuity of the mamillary body with the pons (see Figure 3), but the cephalic flexure insists in obstaculizing that recourse. We in fact proposed the change to “*retromamillary*” already many years before (Puelles and Martínez de la Torre, 1987), and this version has gained some acceptance in the meantime.

I also have been campaigning for a long time for the “single m” orthography of “mammillary.” My rationale is as follows: this word derives from Latin “mamilla,” or nipple, rather than from “mamma,” or breast; in the latter case we would have to say “mammary body,” and nobody does that, irrespective how one subjectively visualizes this brain entity; the problem is that few people nowadays realize that “mamillary” means “nipple-like,” whose root “mamilla” has only one “m”; many classic neuroanatomists possibly knew it, and thus agreed on the “mamillary” orthography. It was mainly post-war colleagues possibly unaware of this etymologic subtlety that spread the wrong orthography. There is a notable review of this orthographic issue by Jones (2011), which is well documented and worth perusing. Jones apparently concludes that it is a matter of usage, and he prefers the one “m” solution, but he weakens his own position by assuming that “mamilla” means “small breast,” in contradiction with my Concise Oxford Dictionary, which says it means “nipple.” I presented the case to the recent FIPAT brain nomenclature committee (ten Donkelaar et al., 2017), but my position was outvoted in favor of conventional usage (search the word under “hypothalamus” in ten Donkelaar et al., 2018). Paraphrasing Kuhlenbeck's citation at the head of this essay, I'll continue spelling “mamillary” with one “m.”

These basal longitudinal domains (all three cutting *de facto* across the columnar hypothalamic unity, but providing conceptual pigeonholes for a number of unexplained potatoes and associated patterning thinking; Puelles, 2017) have double names in our terminology because these domains show some differences between THy and PHy, irrespective of their fundamental molecular and cell typological continuity (these partial differences underpin the idea that THy and PHy belong to different prosomeres if the partial boundaries happen to sum up into a single intrahypothalamic boundary; see Ferran et al., 2015). *Mamillary* and *retromamillary* areas clearly refer to differences between THy and PHy at the ventralmost basal subregion; the anatomists of course already knew that the mamillary body is different in various aspects from the retromamillary area (e.g., projections), though both basically contain excitatory neurons. We now have a neuromeric explanation of why this may be so. Moreover, structures of the *tuberal area* proper, such as infundibulum, median eminence, arcuate nucleus, and neurohypophysis, are only found within basal THy lying under the alar-basal boundary, not more ventrally or more caudally. This also happens with the largest tuberal hypothalamic nucleus, the ventromedial hypothalamic nucleus; it clearly does not extend to the caudal end of the hypothalamus (VM; Figures 10, 13–15). Some other structural features, such as the dorsomedial nucleus, extend instead uniformly across both THy and PHy (see Figure 15C). We thus innovated somewhat by proposing to call *retrotuberal area* the PHy domain placed just behind the THy tuberal area, and dorsal to the periretromamillary area (Tu; RTu; Figures 10, 13–15). While the “*ventromedial”* descriptor in VM is barely acceptable in prosomeric coordinates (the nucleus is “ventral” since it is basal, and it lies next to the periventricular stratum, being thus also “medial”), it would be more precise if the name could be changed to “rostroventromedial nucleus,” because of its restriction to THy, but this results a bit clumsy. It may be useful to remember that Ramón y Cajal (1911) simply named it the “*principal hypothalamic nucleus*.” The topographic name “dorsomedial nucleus” (DM) is instead rather hopeless, since, being partly retrotuberal and partly tuberal, the nucleus lies “*caudal*” to the VM within the PHy and “*ventral*” to the VM within the THy. This occurs because the VM is not born where we see it in the adult; it results from a localized dorsoventral migration stream coming from the dorsalmost tuberal subregion of the THy, which specializes in glutamatergic neurons, as opposed to the underlying DM area, which is rich in GABAergic neurons (Figures 14, 15; some evidence of this migration was shown in Puelles L. et al., 2012a; their Figure 8.26). Without the VM migration, the DM would form a perfectly level longitudinal column through the tuberal/retrotuberal areas, but because of the dorsoventral penetration of the VM it results compressed ventrally (there is no cell mixing at all). The VM also has a peripheral shell of variously migrated cell types, many coming from the alar domains (Díaz et al., 2015).

Further semantic complication emerges when we consider the conventional ventral and dorsal premamillary nuclei (VPM; DPM; Figures 14, 15). The VPM was found to be a migrated blob of excitatory cells stabilizing within the ventralmost terminal part of DM, ventrally to the VM (i.e., within tuberal THy); thanks to various early gene markers, this blob surprisingly was found to originate from the retromamillary area in PHy (see details in Puelles L. et al., 2012a; the migration has been since corroborated experimentally). On the other hand, the DPM, also containing excitatory glutamatergic neurons, belongs to the molecularly distinct perimamillary domain (i.e., restricted to THy; Figures 14, 15) that separates the mamillary/retromamillary regions from the tuberal/retrotuberal region. The main perimamillary derivative represents precisely the population identified as DPM in columnar accounts. It so happens, therefore, that by its retromamillary origin, the VPM is “caudal” to the DPM, but by its adult migrated tuberal position it is “dorsal” to the same. Obviously, this means that in prosomeric coordinates the DPM lies strictly “ventral” to the adult VPM (a terminological disaster). There is no way to save the columnar use of these descriptors, and suggestions for a reasonable solution are invited. I think we should find a nice descriptive term for the migrated “VPM”, possibly such as “ovoid nucleus” (Ov), or something like that, and rename the terminal DPM simply as “perimamillary nucleus” (PM). The perimamillary nucleus and its peduncular periretromamillary companion form a longitudinal band that shares some molecular markers [e.g., *Otp* and *Sim1* expression, also present at the alar paraventricular area (Figure 10). This poses an interesting patterning problem, since these bands are separate but parallel to each other]. However, they also express each other differential markers and also have differential connections according to their respective ascription to THy or PHy. The PRM nucleus found next to the retromamillary area was initially termed “posterior hypothalamus” in the columnar literature (e.g., Bodian, 1939). Unfortunately, this concept of the posterior hypothalamus was later extended arbitrarily into the diencephalic tegmentum as far back as the retroflex tract (PHTh; Figure 1A); this diencephalic tegmental area does not share the hypothalamic *Otp* and *Sim1* markers, implying that this is a case of an inappropriate term, which should be discontinued.

There are many other details that might be discussed on hypothalamic ancient and modern nomenclature and their respective advantages or problems. I think that we have had enough “spice of life” for the present essay. The reader may have gotten a general idea of where we presently are, and knows where he/she may seek further details and explanations, if so desired.

## Author Contributions

The author confirms being the sole contributor of this work and has approved it for publication.

### Conflict of Interest Statement

The author declares that the research was conducted in the absence of any commercial or financial relationships that could be construed as a potential conflict of interest.

## Abbreviations

3: oculomotor nerve
4: trochlear nerve
5: trigeminal nerve root
6: abducens nerve root
7: facial nerve root
8: cochleovestibular nerve root
ABasM: median anterobasal nucleus
ABasW: anterobasal wing
ac: anterior commissure
AC: nucleus of the anterior commissure
AD: dorsal alar region
AH: anterior hypothalamic nucleus
AHP: peduncular (posterior) part of anterior hypothalamic nucleus
AL: lateral alar region
AP: alar plate
APT: anterior pretectal nucleus
Arc: arcuate nucleus
ArcW: arcuate wing
av: anteroventral thalamic area
AVL: ventrolateral alar region
BI: intermediate basal region
BIC: brachium of the inferior colliculus
BL: lateral basal region
BM: medial basal region
BP: basal plate
BSC: brachium of the superior colliculus
BST: bed nucleus of the stria terminalis (supracapsular)
c.p.: posterior commissure
Cb: cerebellum
Cd: caudate tail
CERVEL.: cerebellum
ch: chorioidal roof
CIC: central nucleus of inferior colliculus
Cn: cuneate nucleus
CnG: central gray
Co: cochlear column
com.post: posterior commissure
CoPT: commissural pretectum
CPa: central part of main paraventricular nucleus
DCIC: dorsal nucleus of inferior colliculus
Dg: diagonal area
DHyB: diencephalo-hypothalamic boundary
Di: diencephalon
Dien: diencephalon
Dk: nucleus of Darkschewitsch
DLTg: dorsolateral tegmental nucleus
DMcP: Dorsomedial core, peduncular part
DMcT: dorsomedial core area, terminal part
DMsP: dorsomedial shell area, peduncular part
DMsT: dorsomedial shell area, terminal part
DPa: dorsal part of main paraventricular nucleus
DpG: deep (central) gray
DPM: dorsal premamillary nucleus
DPML: lateral stratum of DPM
DR: dorsal raphe nucleus
DTg: dorsal tegmental nucleus
DTh: dorsal thalamus
E: epiphysis
e.e.: epiphyseal evagination
e.x.: habenular commissure
ECIC: external nucleus of inferior colliculus
em.th: eminentia thalami
ep: epiphysis
EPIPH: epiphysis
ETh: epithalamus
f.r.: fasciculus retroflexus
FP: floor plate
fx: fornix tract
H: habenula
h.s-t.r.: habenulo-subthalamic ridge (zona limitans)
hab: habenula
HB: habenula
Hb: habenula
hp1-hp2: hypothalamo-telencephalic prosomeres 1-2
HTh: hypothalamus
hy.: hypothalamus
IC: inferior colliculus
IC: interstitial nucleus of Cajal
ICbP: inferior cerebellar peduncle
ICo: intercollicular nucleus
IF: interpeduncular fossa
InG: intermediate gray
IP: interpeduncular nucleus
IR: rostral interstitial nucleus
JcPT: juxtacommissural pretectum
LA: lateral anterior nucleus
lc: lamina cornea (BST)
LCh: laterochiasmatic nucleus
LCo: locus coeruleus
LG: lateral geniculate nucleus
LGN: lateral geniculate nucleus
LiC: nucleus linearis caudalis
LLD: dorsal lateral lemniscal nucleus
LLV: ventral lateral lemniscal nucleus
LM: lateral mamillary nucleus
m: mamillary body
m1-m2: mesencephalic prosomeres or mesomeres 1-2
ma: mamillary body
Mam: mamillary body
MB: mamillary body
MCbP: middle cerebellar peduncle
MDB: mesencephalo-diencephalic boundary
ME: median eminence
Med: medulla
Mes: mesencephalon
mesV: mesencephalic trigeminal nucleus
MG: medial geniculate nucleus
mge: medial ganglionic eminence
MHB: midbrain-hindbrain boundary
MM: medial mamillary nucleus (ventral part)
MPO: medial preoptic nucleus
MT: medial terminal nucleus
mtg: mamillotegmental tract
mth: mamillothalamic tract
MTu: medial tuberal nucleus
n.h.: nucleus habenulae
NA: nuclei of amygdala (medial)
NH: neurohypophysis
NHy: neurohypophysis
och: optic chiasma
OPT: olivary pretectal nucleus
ot: optic tract
P.AL.: alar plate
P.BAS: basal plate
p.d.th.m.: pars dorsalis thalami (middle part)
p.i.d.: pars intermedia diencephali (pretectum)
p.v.th.: pars ventralis thalami
p1–p3: diencephalic prosomeres 1-3
p1PAG: pretectal periaqueductal gray
p1Tg: pretectal tegmentum
p2Tg: thalamic tegmentum
p3Tg: prethalamic tegmentum
Pa: paraventricular hypothalamic area
pa: paraventricular hypothalamic area
PAG: periaqueductal area
Pal: pallidum
pallial A: pallial amygdala
PaR: pararubral nucleus
PB: parabrachial nucleus
PBas: posterobasal nucleus
PBG: parabigeminal nucleus
pc: posterior commissure
pc: posterior commissure
PCMc: magnocellular nucleus of the posterior commissure
PCPc: parvocellular nucleus of the posterior commissure
PcPT: precommissural pretectum
pd: posterodorsal thalamic area
Ped: peduncle
PHTh: posterior hypothalamus
PHy: peduncular hypothalamus
PL.V.: floor plate
PLTg: posterolateral tegmental nucleus
pm: perimamillary area
PM: perimamillary nucleus (dorsal premamillary n.)
Poa: preoptic area
POA: preoptic area
poa: preoptic area
PPa: peduncular paraventricular area
PPn: pedunculopontine nucleus
PreIsth: preisthmus
pret: pretectum
prm: periretromamillary area
PRM: periretromamillary nucleus
PRML: lateral stratum of PRM
PRuTg: prerubral tegmentum
PSPaZ: peduncular subparaventricular zone
PT: pretectum
pt: pretectum
PTh: prethalamus
pth: prethalamus
PThE: prethalamic eminence
pthe: prethalamic eminence
R: rhombencephalon
r.m.: mamillary recess
r0-r11: rhombomeres 0-11
rf: retroflex tract
rf: retroflex tract
Rh: rhombencephalon
rm: retromamillary area
RM: retromamillary area
RMC: magnocellular red nucleus
RML: lateral retromamillary nucleus
RMM: medial retromamillary nucleus
RP: roof plate
RPa: rostral paraventricular area
RPC: parvocellular red nucleus
Rt: reticular nucleus (prethalamus)
rtu: retrotuberal area
RTuV: ventral retrotuberal area
RuMc: magnocellular red nucleus
RuPc: parvocellular red nucleus
S.TH.M.: sulcus thalami medius
S.THAL.VEN.: sulcus thalami ventralis
SbPO: subpreoptic nucleus
SC: spinal cord
SC: superior colliculus
SCbP: superior cerebellar peduncle
SCH: suprachiasmatic nucleus
SCH: suprachiasmatic nucleus
SDD: sulcus diencephali dorsalis
SDM: sulcus diencephali medius
SdV: descending sensory trigeminal nucleus
SDV: sulcus diencephali ventralis
se: septum
Sec.Pros.: secondary prosencephalon
SL: sulcus limitans
SNR: substantia nigra
SPa: subparaventricular hypothalamic area
spa: subparaventricular hypothalamic area
SpV: principal sensory trigeminal nucleus
St: striatum
s-t.: subthalamus
std: sulcus thalami dorsalis
STh: subthalamic nucleus
stm: sulcus thalami medius
stv: sulcus thalami ventralis
SubB: subbrachial nucleus
subpall. A: subpallial amygdala
tc: tectal commissure
Tel: telencephalon
TG: tectal gray
tg: tegmentum
tgc: tectal gray commissure
Tgp (Fi): fimbria hippocampi
TGPC: tectal gray paracommissural nucleus
Th: thalamus
TH: thalamus
th: thalamus
th.1: thalamic bulge 1 (prethalamus)
th.2: thalamic bulge 2 (thalamus)
TH.D: dorsal thalamus
TH.V.: ventral thalamus
THy: terminal hypothalamus
TPa: terminal paraventricular area
TPCD: tectal paracommissural dorsal nucleus
TPCV: tectal paracommissural ventral nucleus
tpt: tractus peduncularis transversus
tpth: taenia prethalamica
TSO: terminal supraoptic nucleus
TSPaZ: terminal subparaventricular zone
tst: taenia striae terminalis
tth: taenia thalami
Tu: tuberal area
tu: tuberal area
TuSbO: tuberal suboptic nucleus
TuV: tuberal ventral area
U: uncus
v.t.: velum transversum
VEL.TR.: velum transversum
Vest: vestibular column
VM: ventromedial nucleus
VMH: ventromedial hypothalamic nucleus
VMs: shell of ventromedial nucleus
VPa: ventral part of main paraventricular nucleus
VPM: ventral premamillary nucleus
VTA: ventral tegmental area
VTg: ventral tegmental nucleus
VTh: ventral thalamus
zi: zona incerta (prethalamus)
ZIR: rostral zona incerta
zli: zona limitans intrathalamica
Zr: reticular nucleus.
